# Genome-Wide Histone Acetylation Is Altered in a Transgenic Mouse Model of Huntington's Disease

**DOI:** 10.1371/journal.pone.0041423

**Published:** 2012-07-27

**Authors:** Karen N. McFarland, Sudeshna Das, Ting Ting Sun, Dmitri Leyfer, Eva Xia, Gavin R. Sangrey, Alexandre Kuhn, Ruth Luthi-Carter, Timothy W. Clark, Ghazaleh Sadri-Vakili, Jang-Ho J. Cha

**Affiliations:** 1 Department of Neurology, MassGeneral Institute for Neurodegenerative Disease, Massachusetts General Hospital, Charlestown, Massachusetts, United States of America; 2 MIND Informatics, MassGeneral Institute for Neurodegenerative Disease, Massachusetts General Hospital, Cambridge, Massachusetts, United States of America; 3 Brain Mind Institute, Ecole Polytechnique Fédérale de Lausanne, Lausanne, Switzerland; Radboud University, The Netherlands

## Abstract

In Huntington's disease (HD; MIM ID #143100), a fatal neurodegenerative disorder, transcriptional dysregulation is a key pathogenic feature. Histone modifications are altered in multiple cellular and animal models of HD suggesting a potential mechanism for the observed changes in transcriptional levels. In particular, previous work has suggested an important link between decreased histone acetylation, particularly acetylated histone H3 (AcH3; H3K9K14ac), and downregulated gene expression. However, the question remains whether changes in histone modifications correlate with transcriptional abnormalities across the entire transcriptome. Using chromatin immunoprecipitation paired with microarray hybridization (ChIP-chip), we interrogated AcH3-gene interactions genome-wide in striata of 12-week old wild-type (WT) and transgenic (TG) R6/2 mice, an HD mouse model, and correlated these interactions with gene expression levels. At the level of the individual gene, we found decreases in the number of sites occupied by AcH3 in the TG striatum. In addition, the total number of genes bound by AcH3 was decreased. Surprisingly, the loss of AcH3 binding sites occurred within the coding regions of the genes rather than at the promoter region. We also found that the presence of AcH3 at any location within a gene strongly correlated with the presence of its transcript in both WT and TG striatum. In the TG striatum, treatment with histone deacetylase (HDAC) inhibitors increased global AcH3 levels with concomitant increases in transcript levels; however, AcH3 binding at select gene loci increased only slightly. This study demonstrates that histone H3 acetylation at lysine residues 9 and 14 and active gene expression are intimately tied in the rodent brain, and that this fundamental relationship remains unchanged in an HD mouse model despite genome-wide decreases in histone H3 acetylation.

## Introduction

Huntington's disease (HD) is a progressive neurodegenerative disorder resulting from a trinucleotide CAG repeat expansion in the *Huntingtin* gene [1]. Pathologically, HD is characterized by a preponderance of neuronal death in the striatum (caudate-putamen). HD patients suffer a triad of movement, cognitive and behavioral issues which steadily worsen throughout the course of the disease [2]. There are currently no effective treatments and the key pathogenic mechanisms that are responsible for the striatal vulnerability leading to the progressive neurodegeneration are unknown.

Transcriptional dysregulation is a characteristic of the disease process in human patients and is faithfully recapitulated in multiple animal and cellular models [3]. Abnormalities in transcription occur prior to the onset of symptoms and are accompanied by changes in histone acetylation, ubiquitylation and methylation [4]–[9]. However, whether changes in histone modifications result in the transcriptional abnormalities remains a largely unanswered question.

In particular, acetylation of the N-terminal tail of histone H3 is an activating mark for gene expression [10], [11], and increases in histone acetylation precede and facilitate increased transcriptional activity [12], [13]. In HD, levels of acetylated histone H3 (AcH3) associated with downregulated genes are decreased [6]. In an HD cell line and transgenic HD mouse model, mRNA abnormalities were reversed by treatment with inhibitors of histone deacetylases (HDAC), the family of enzymes that remove acetyl groups from histone tails, with concomitant increases in global histone H3 acetylation [6]. Furthermore, decreases in histone acetylation and mRNA levels in the HD cell line can be mimicked in wild-type cells by inhibiting histone acetyltransferases (HATs), enzymes that catalyze the removal of acetyl groups from histone proteins [6]. These results, though limited to a few genes, suggest that decreasing histone acetylation at gene loci is necessary and sufficient for concomitant decreases in mRNA levels. Subsequently, HDAC inhibition is currently being investigated as potential therapeutic intervention for HD as well as other neurodegenerative disorders [14], [15]. However, the relationship between histone acetylation and gene expression has not been studied at the level of the whole genome in the mammalian brain. Furthermore, it is not currently known whether this relationship is altered in the HD brain. While we do know that global levels of histone acetylation do not correspond to histones at specific gene loci [6], it is unknown if the genome-wide distribution of histone acetylation is altered in HD or if the genomic distribution of histone acetylation accounts for gene expression abnormalities.

We used a genome-wide approach to capture acetylated histone H3 K9/K14 (AcH3)-DNA interactions and interrogated the chromatin immunoprecipitation products on DNA microarrays (ChIP-chip) to determine genomic locations of AcH3 in the striatum of wild-type (WT) and transgenic (TG) R6/2 mice, a mouse model for HD [16]. This analysis allowed us to examine AcH3-binding events at gene loci in WT and TG brain and then to draw comparisons of differential binding states. Using a previously published gene expression microarray dataset of R6/2 mice at the same age [17], we then correlated AcH3-bound gene loci with steady-state gene expression levels in the striatum.

We find that AcH3-gene associations are decreased genome-wide in the TG R6/2 striatum. In both WT and TG striata, AcH3 is predominately located within the coding region of the gene as opposed to the upstream promoter region. Additionally, AcH3 association at gene loci strongly correlates with genes that are actively expressed in both WT and TG striatum, suggesting that the fundamental relationship between histone H3 acetylation and gene transcription is not altered in transgenic mice. Furthermore, HDAC inhibitor treatment of TG R6/2 mice reverses transcriptional abnormalities of individual gene and increases global levels of histone H3 acetylation but AcH3 levels at specific gene loci are increased for only a handful of the tested genes. Thus, these data indicate that the overall chromatin environment surrounding a gene is more influential for the transcriptional activity of that gene. These data reveal for the first time the strong correlation between transcription and histone H3 acetylation in the brain and demonstrate that this fundamental relationship is unchanged in an HD mouse model.

## Results

DNA products of acetylated histone H3 K9/K14 (AcH3) chromatin immunoprecipitations (ChIP) from WT and TG striatum were labeled and hybridized on promoter microarrays consisting of 60-mer oligonucleotide probes that comprise over 18,000 genes, each of which is represented across multiple probes. Though the microarray used was a genomic promoter microarray, thus representing only a fraction of the entire genome as a tiling microarray would, probes were distributed across the entire gene region and were not restricted to the upstream region of the core promoter immediately near the transcriptional start site of the gene.

### AcH3 levels are decreased at gene loci in R6/2 TG striatum

**Figure 1:**
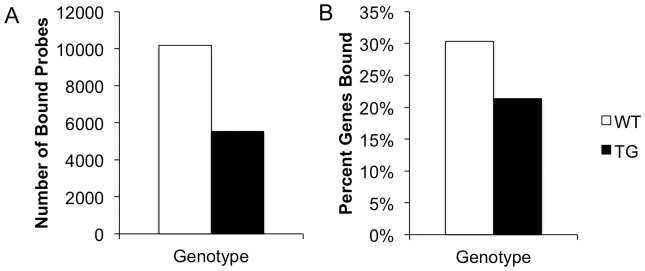
AcH3 interactions at gene loci are decreased in TG striata. (A) The number of AcH3-bound probes in WT (white bars) and TG (black bars) striata. (B) Percent of total genes bound by AcH3 in WT and TG striata.

**Figure 2:**
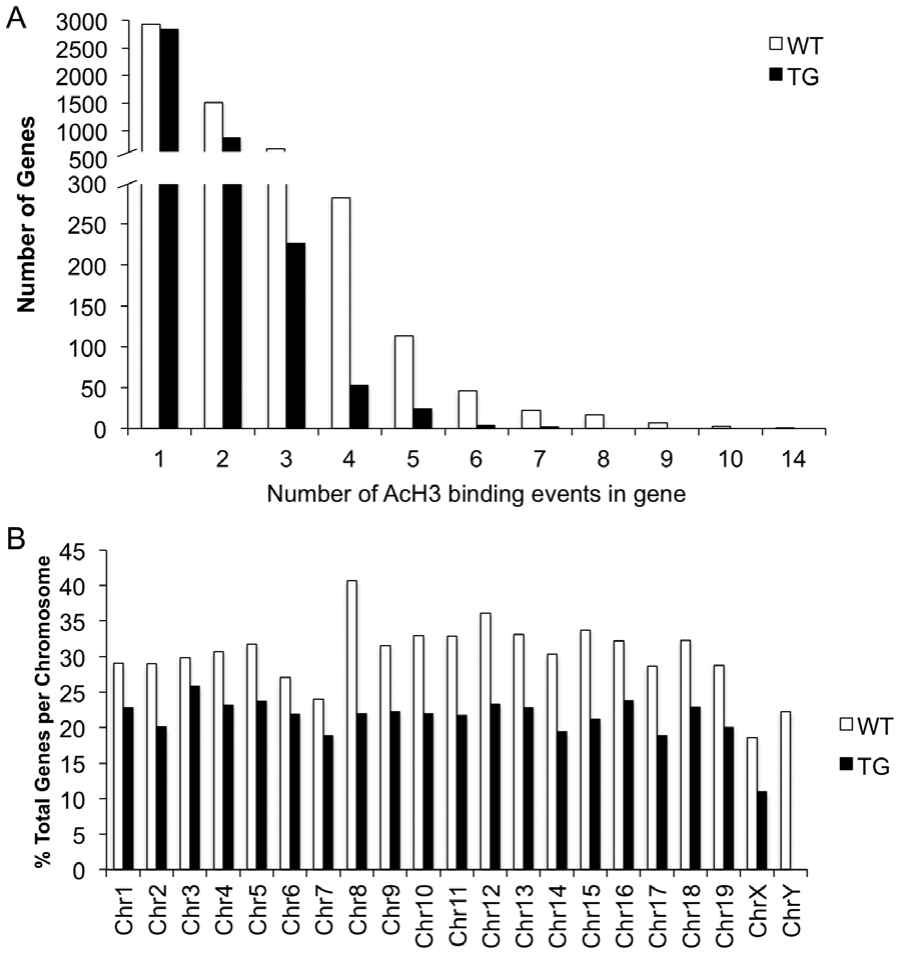
AcH3 binding patterns within a gene and across chromosomes are diminished in TG striata. (A) Distribution of the number of AcH3 binding events in WT and TG striata expressed as probes bound per gene. (B) Distribution of AcH3-gene associations for each chromosome in WT and TG striata expressed as a percentage of AcH3-bound genes per total genes on each chromosome. White bars, WT; black bars, TG.

In the wild-type (WT) striatum, we find that AcH3 is present at 10,187 probes or 2.1% of total probes (Figure 1A). These AcH3-bound probes are distributed across 5,606 genes representing 29.6% of genes on the microarray (Figure 1B). At a single gene locus, AcH3 binds at a maximum of 14 individual probes (Figure 2A).

In the transgenic (TG) R6/2 striatum, we find that AcH3 is bound to 5,542 probes representing 1.2% of total probes, a level that is 54.4% of that seen in WT littermates (Figure 1A). In the TG striatum, these AcH3-bound probes are distributed across 4,011 genes (21.2% of total genes, 71.5% of WT littermates, Figure 1B) and bind a maximum of 7 probes per gene (Figure 2A).

### AcH3 binding is distributed evenly amongst and along chromosomes

To determine the genome-wide distribution of AcH3 bound genes, we first determined the total number of annotated genes represented on the microarray for each chromosome. We then calculated the percentage of AcH3-bound genes per chromosome. As seen in Figure 2B, AcH3 binding events in the WT striatum are distributed relatively evenly across all chromosomes. In the TG striatum, decreases in histone H3 acetylation were seen across all chromosomes and averaged to approximately 70% of WT levels with the exclusion of chromosome Y which is represented by only 9 genes on the Agilent array. The largest decreases were seen on chromosome 8 where AcH3-gene associations were 54% of WT and the smallest were on chromosome 3 where associations in TG is 87% of WT levels.

**Figure 3:**
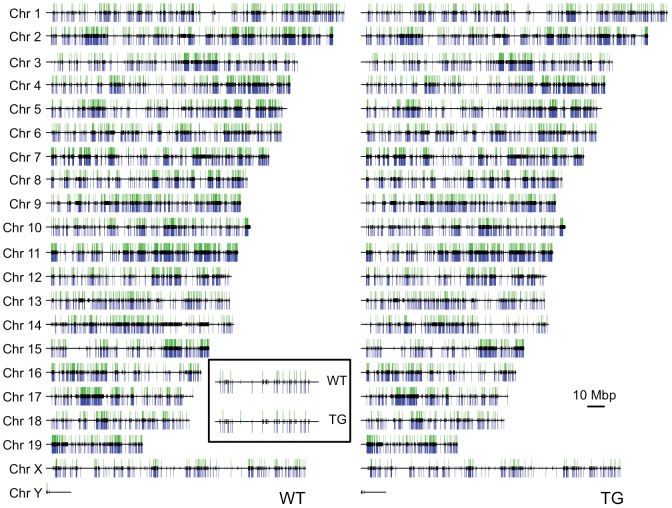
AcH3-gene associations are distributed across the genome and correlate with expressed genes. Localization of AcH3-gene interactions (green) along each chromosome and are co-localized with steady-state gene expression (blue) in WT (left) and TG striata (right). Horizontal black line, length of chromosome; Vertical black lines, locations of genes. Inset, a closer view of Chromosome 1 from 90574275 bp–93452450 bp.

Visualization of AcH3 binding events along each chromosome clearly demonstrates that AcH3-gene associations are evenly distributed in WT (Figure 3A). Any clustering of AcH3 binding is primarily due to the high density of genes in these regions (note the clustering of black lines representing gene locations, in Figure 3). Acetylated histone H3 distribution patterns are similar between chromosomes and are not in highly isolated patches along each individual chromosome. In the TG striatum, we see a similar pattern as well in spite of reduced AcH3 levels (Figure 3B). Thus, despite overall decreases in AcH3 levels in the TG striatum, this decrease does not appear to be biased towards any chromosome region.

### AcH3 is localized within the coding region of the gene

**Figure 4:**
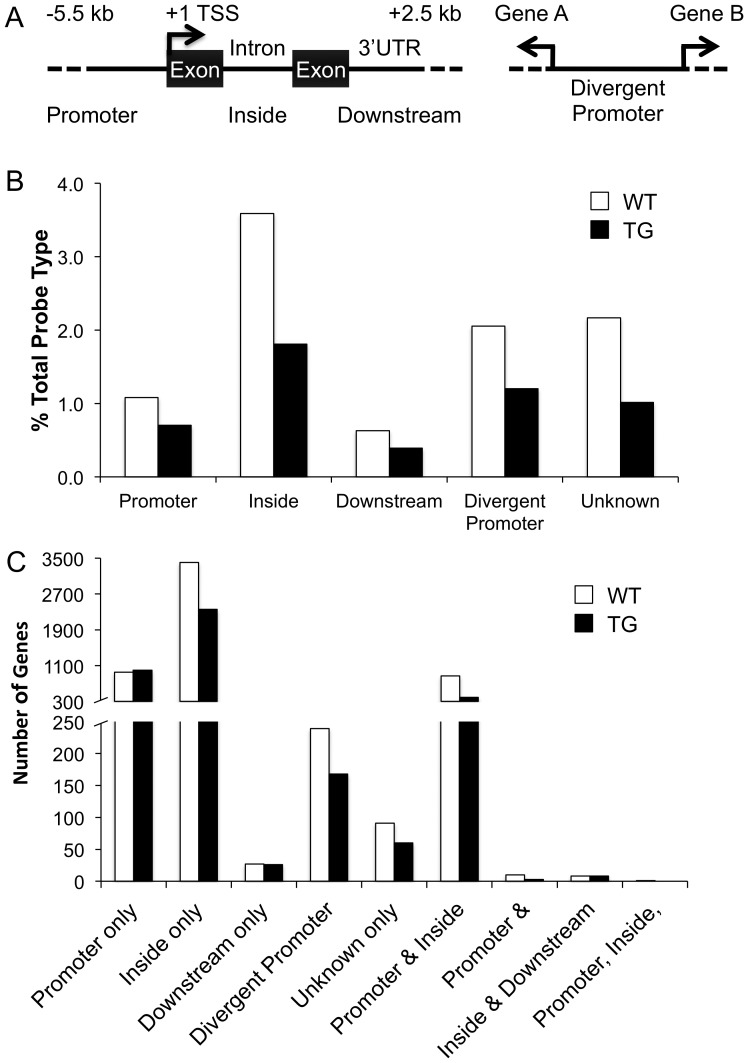
AcH3 localization is found downstream of transcriptional start sites within the gene. (A) Schematic indicating the location of probes as defined by Agilent annotation. Promoter = upstream of transcriptional start site; Inside = within coding region of gene; Downstream = downstream of coding region; Divergent promoter = located within the upstream region of two genes transcribed in opposite directions; Unknown = unannotated. (B) Locations of probes within the gene expressed as percent bound of total probe type within the microarray. (C) Numbers of genes bound as expressed by AcH3-bound locationsWhite bars, WT; black bars, TG.

To determine where AcH3-bound probes are located within the gene, we examined the bound probes by probe type as defined by Agilent's annotation—Promoter, Inside, Downstream, Divergent Promoter and Unknown (see Figure 4A). Here, we examined the number of bound probes as a percentage of total probe type as the promoter and inside probes were predominately represented on the microarray. In the WT striatum, we find that of the 10,187 AcH3-bound probes, AcH3 localization within the gene is unevenly distributed and is found primarily bound to inside probes, which encompasses the coding region of the gene, as well as a smaller percentage of promoter probes (Figure 4B). AcH3-bound probes in the TG striatum follow a similar pattern as in the WT striatum but are decreased with levels ranging from 45% to 67% percent of WT (Figure 4B).

Individual genes can have unique AcH3 binding signatures with AcH3 present only at one region (i.e., exclusively at promoter regions) or a combination of regions (i.e., promoter and inside regions together). Accordingly, we analyzed AcH3-bound genes for these binding signatures and find that the majority of AcH3-bound genes have AcH3 exclusively within the inside coding region (Figure 4C). A smaller number of genes have AcH3 bound solely at the promoter region and an even smaller group of genes have AcH3 bound at both the promoter and inside region together or in the divergent promoter only. In this analysis, we unexpectedly found that decreases in AcH3-bound genes in the TG striatum did not occur evenly for all categories. AcH3-gene associations within “Promoter Only”, “Downstream Only” as well as “Inside & Downstream” categories remained at levels similar to WT. AcH3 bound at “Promoter & Inside” regions in addition to “Promoter & Downstream” regions were dramatically reduced to 45% and 30% of WT levels, respectively. The remaining categories of AcH3-bound genes were reduced to levels ranging from 66 to 70% of WT.

### Individual genes are differentially acetylated between WT and TG striatum

We next examined differential histone H3 acetylation patterns between WT and TG striata at individual genes. Here, we utilized the results from our composite score analysis. This score allows us to quantitatively determine levels of AcH3 levels at gene loci by accounting for the number of probes bound at the gene but also the signal intensity of these probes. With this method we identified genes with decreased histone H3 acetylation in TG (Table 1) as well as a lesser number of genes with increased histone H3 acetylation in TG (Table 2).

**Table 1:** The top genes with decreased histone H3 acetylation in TG striata.

Rank	Gene Symbol	Gene Name	Chromosome	No of WT Probes	No of TG Probes	Composite Score
1	*Zfp469/Tnrc18*	*zinc finger protein 469; trinucleotide repeat containing protein 18*	chr5	14	6	−11.33
2	*Per1*	*period homolog 1 (Drosophila)*	chr11	8	2	−10.60
3	*BC057627/Zc3h4*	*zinc finger CCCH-type containing type 4*	chr7	10	2	−10.53
4	*Pik3r1*	*phosphatidylinositol 3-kinase, regulatory subunit, polypeptide 1 (p85 alpha)*	chr13	9	3	−9.17
5	*Homer1*	*homer homolog 1 (Drosophila)*	chr13	9	2	−8.59
6	*mmu-mir-124a-2*	*microRNA*	chr3	9	1	−8.53
7	*Lrrtm2*	*leucine rich repeat transmembrane neuronal 2*	chr18	9	4	−8.02
8	*Cpeb4*	*cytoplasmic polyadenylation element binding protein 4*	chr11	10	5	−7.90
9	*Gpm6a*	*glycoprotein m6a*	chr8	8	1	−7.81
10	*Chn1*	*chimerin (chimaerin) 1*	chr2	9	3	−7.67
11	*Elmod1*	*ELMO domain containing 1*	chr9	7	1	−7.65
12	*Epc1*	*enhancer of polycomb homolog 1*	chr18	10	5	−7.49
13	*Stmn4*	*stathmin-like 4*	chr14	6	0	−7.41
14	*Cacna2d3*	*calcium channel, voltage-dependent, alpha2/delta subunit 3*	chr14	6	0	−7.39
15	*Rtn1*	*reticulon 1*	chr12	8	2	−7.32
16	*Negr1*	*neuronal growth regulator 1*	chr3	8	3	−7.12
17	*Sec14l1*	*SEC14-like 1 (S. cerevisiae)*	chr11	6	1	−7.03
18	*Mgrn1*	*mahogunin, ring finger 1*	chr16	7	2	−7.00
19	*Neto1*	*neuropilin (NRP) and tolloid (TLL)-like 1*	chr18	7	3	−6.97
20	*Eif3s7/Eif3d*	*eukaryotic translation initiation factor 3, subunit D*	chr15	8	2	−6.95
21	*Grik2*	*glutamate receptor, ionotropic, kainate 2 (beta 2)*	chr10	7	3	−6.91
22	*Bat2*	*HLA-B associated transcript 2*	chr17	8	5	−6.89
23	*Kcnj2*	*potassium inwardly-rectifying channel, subfamily J, member 2*	chr11	8	4	−6.81
24	*Zfhx2*	*zinc finger homeobox 2*	chr14	7	3	−6.75
25	*9430023L20Rik*	*RIKEN cDNA 9430023L20 gene*	chr15	7	1	−6.74
26	*Rab30*	*RAB30, member RAS oncogene family*	chr7	8	4	−6.63
27	*mmu-mir-124a-1*	*microRNA*	chr14	8	5	−6.61
28	*Mark2*	*MAP/microtubule affinity-regulating kinase 2*	chr19	6	2	−6.58
29	*Cugbp2*	*CUGBP, Elav-like family member 2*	chr2	8	2	−6.55
30	*Rgs9*	*regulator of G-protein signaling 9*	chr11	5	1	−6.50

Gene symbol annotation for these genes was checked with NCBI's Entrez Gene database (http://www.ncbi.nlm.nih.gov/sites/entrez). In the instances where gene annotation has been updated, we have left the original Agilent annotation intact and added the updated information subsequent to this in the form of Original Gene Symbol/Updated Gene Symbol.

**Table 2:** The top genes with increased histone H3 acetylation in TG striata.

Rank	Gene Symbol	Gene Name	Chromosome	No of WT Probes	No of TG Probes	Composite Score
1	*Olfr1446*	*olfactory receptor 1446*	chr19	0	2	4.03
2	*A430060F13Rik*	*RIKEN cDNA A430060F13 gene*	chr11	0	1	3.63
3	*Slc26a6*	*solute carrier family 26, member 6*	chr9	0	1	3.13
4	*Wif1*	*Wnt inhibitory factor 1*	chr10	0	1	3.05
5	*Zfp367*	*zinc finger protein 367*	chr13	1	2	2.99
6	*A130007F10Rik/Dcaf12l2*	*DDB1 and CUL4 associated factor 12-like 2*	chrX	0	3	2.67
7	*Ankrd54/C730048E16Rik-Eif3s6ip/Eif3l*	*ankyrin repeat domain 54- eukaryotic translation initiation factor 3, subunit L*	chr15	0	1	2.30
8	*A730017C20Rik*	*RIKEN cDNA A730017C20 gene*	chr18	0	1	2.14
9	*Gprc6a*	*G protein-coupled receptor, family C, group 6, member A*	chr10	0	1	2.06
10	*BC089469/2210408I21Rik*	*RIKEN cDNA 2210408I21 gene*	chr13	0	1	1.77
11	*Olfr1500*	*olfactory receptor 1500*	chr19	0	1	1.75
12	*Tbc1d5*	*TBC1 domain family, member 5*	chr17	1	1	1.73
13	*Mrpl16*	*mitochondrial ribosomal protein L16*	chr19	0	1	1.71
14	*9230117N10Rik/Il33*	*interleukin 33*	chr19	0	1	1.60
15	*Tmem16a/Ano1*	*anoctamin 1, calcium activated chloride channel*	chr7	0	2	1.58
16	*Vkorc1l1*	*vitamin K epoxide reductase complex, subunit 1-like 1*	chr5	0	2	1.53
17	*Xrn1*	*5′-3′ exoribonuclease 1*	chr9	1	2	1.50
18	*Slc25a37*	*solute carrier family 25, member 37*	chr14	0	2	1.50
19	*Fgf9*	*fibroblast growth factor 9*	chr14	0	2	1.49
20	*Trim42*	*tripartite motif-containing 42*	chr9	0	1	1.47
21	*Olfr735*	*olfactory receptor 735*	chr14	0	1	1.42
22	*Kcnh6*	*potassium voltage-gated channel, subfamily H (eag-related), member 6*	chr11	0	2	1.41
23	*1110051M20Rik*	*RIKEN cDNA 1110051M20 gene*	chr2	0	2	1.39
24	*ENSMUST00000078552*	*Ensembl gene BX119986.8-201*	chrX	0	2	1.39
25	*Arhgap17*	*Rho GTPase activating protein 17*	chr7	0	2	1.37
26	*Chuk*	*conserved helix-loop-helix ubiquitous kinase*	chr19	2	4	1.37
27	*BC042698/Dennd1b*	*DENN/MADD domain containing 1B*	chr1	0	2	1.37
28	*Map3k3*	*mitogen-activated protein kinase kinase kinase 3*	chr11	0	2	1.37
29	*Terf2*	*telomeric repeat binding factor 2*	chr8	0	2	1.34
30	*Pisd*	*phosphatidylserine decarboxylase*	chr5	0	2	1.33

Gene symbol annotation for these genes was checked with NCBI's Entrez Gene database (http://www.ncbi.nlm.nih.gov/sites/entrez). In the instances where gene annotation has been updated, we have left the original Agilent annotation intact and added the updated information subsequent to this in the form of Original Gene Symbol/Updated Gene Symbol.

**Figure 5:**
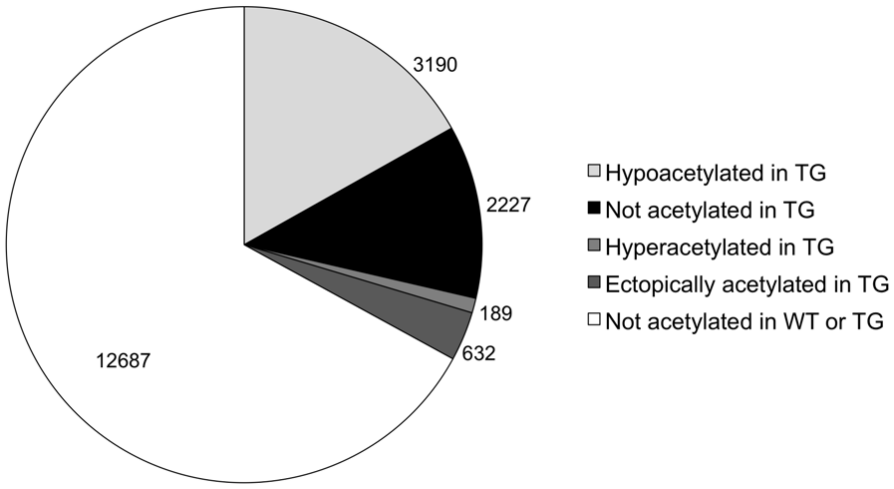
Differential histone H3 acetylation within WT and TG striata. **Hypoacetyled in Tg** (light grey), gene is bound by AcH3 in both WT and TG but binding is decreased in TG; **Not acetylated in TG** (black), AcH3 is associated with the gene only in the WT state; **Hyperacetylated in TG** (medium grey), gene is bound by AcH3 in both WT and TG but binding is increased in TG; **Ectopically acetylated in TG** (dark grey), AcH3 is associated with the gene only in the TG state; **Not acetylated**, gene does not have AcH3 associated in either WT or TG states.

Using the composite score, we further categorized four types of differential AcH3-gene binding: 1) “Not acetylated in TG”, where AcH3 is bound at the gene in WT, but not in TG; 2) “Hypoacetylated in TG”, where AcH3 is bound at the gene in both WT and TG but to a lesser degree in TG; 3) “Ectopically acetylated in TG”, where AcH3 is bound at the gene in TG, but not in WT; and 4) “Hyperacetylated in TG”, where AcH3 is bound at the gene in both WT and TG, but to a greater degree in TG (Figure 5). A large number of genes fell into a fifth category where AcH3 was not present at the gene in either WT or TG striata. In this comparison between WT and TG striata, we find differential AcH3 levels are predominately decreased with “Hypoacetylated in TG” and “Not acetylated in TG” categories accounting for greater than 85% of the changes seen. Surprisingly, in spite of globally decreased levels of AcH3-gene interactions in TG striatum, we also found genes with increased histone acetylation at specific gene loci in TG striatum which accounted for the remaining 13.2% differentially acetylated genes.

We analyzed the genes within these four categories (“Hypoacetylated in TG”, “Not acetylated in TG”, “Hyperacetylated in TG” and “Ectopically acetylated in TG”) for gene ontology (GO) functional annotation term clustering to identify similar GO terms associated with these gene lists (Tables S3, S4, S5, S6). Annotation clustering ranks groups of functionally related annotations [18]. For our analysis, we included functional groups with enrichment scores of greater than 1.3 as this corresponds to a non-log value of 0.05. Not surprisingly as it was the most numerous list, “hypoacetylated in TG” genes were the most densely populated list of annotation clusters. “Not acetylated in TG” genes also had a number of annotation clusters and only a handful of these clusters were shared between these two gene lists (e.g., protein localization or mRNA processing). “Hyperacetylated in TG” and “ectopically acetylated in TG” gene lists each had only two annotation clusters with an enrichment score of 1.3 or greater. These clusters included genes involved in transcription (cluster 1, hyperacetylated in TG) and protein localization (cluster 2, hyperacetylated in TG) as well as gonad development and reproductive processes (cluster 1, ectopically acetylated in TG) and cell motility and migration (cluster 2, ectopically acetylated in TG).

### Single Gene ChIP confirmation

We then ranked genes using the composite score to determine those with the greatest changes in histone H3 acetylation levels between WT and TG. Genes with the greatest decreases in histone H3 acetylation are listed in Table 1. Within this table, the majority of these genes are classified as hypoacetylated given that the probes for these gene are bound in both WT and TG with a reduced number of probes and reduced signal (data not shown) for that gene bound in TG striata. Using the same approach, we ranked genes whose acetylation levels are increased in TG striata (Table 2).

**Figure 6:**
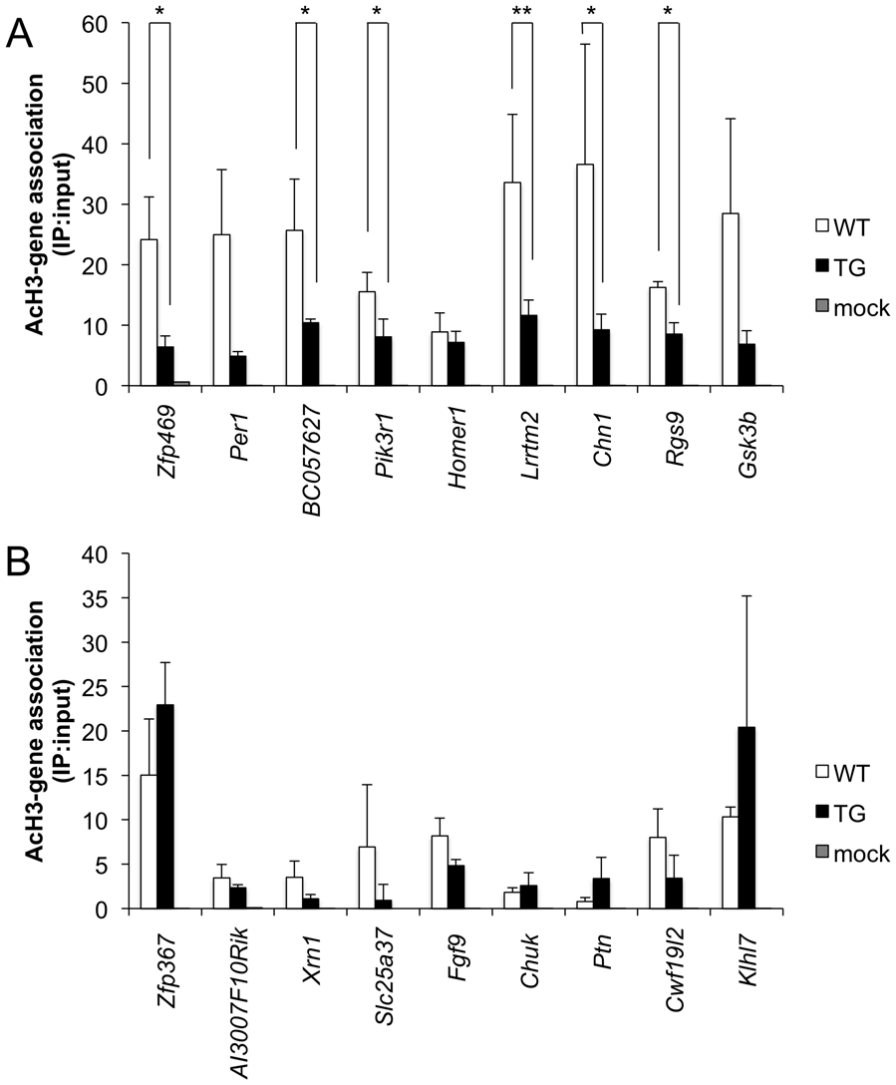
Single gene ChIP confirmation of differentially acetylated genes. (A) Genes with decreased acetylation as predicted by their composite score. (B) Genes with increased acetylation as predicted by their composite score. The order of the genes from left-right indicates their relative ranking by composite score. *, p-value<0.05; **, p-value<0.01. White bars, WT; black bars, TG; grey bars, mock condition. Error bars are standard deviation.

We then designed gene-specific primers for single-gene ChIP testing of hypoacetylated gene loci. The primer pairs were based on the microarray annotation supplied by Agilent and amplified genomic sequences surrounding differentially bound probes within the gene loci. Of those genes tested, we confirmed that there is a decrease in AcH3-association at these gene loci in TG striata (Figure 6A). We performed a similar analysis of hyperacetylated genes—AcH3 association was increased in the TG striatum at only a few of the genes tested (Figure 6B); however, these increases did not reach a level of significance.

### Histone H3 acetylation at gene loci predicts active gene transcription

We further investigated the relationship between histone H3 acetylation at the gene and expression of that gene. Here, we wanted to determine whether the presence of histone H3 acetylation at the gene loci correlates to the presence of the gene's transcript upon analysis of gene expression microarrays. For this, we confined our analysis to the subset of nearly 13,000 genes that are common to both the Agilent ChIP-chip microarrays as well as the Affymetrix expression microarrays.

We examined the association between histone H3 acetylation at gene loci and the presence of the gene transcript in WT and TG striatum (Tables 3 & 4, respectively). We simplified this analysis by qualitatively categorizing each gene as bound or not bound by AcH3 and its transcript as expressed or not expressed. We find that in both WT and TG striatum, the majority of AcH3-associated genes also have a gene transcript present indicating active gene transcription. In the WT striatum, 93% of genes associated with acetylated histone H3 are transcribed (Table 3) revealing a significant association between the presence of histone H3 acetylation at the gene locus and the presence of its transcript (odds ratio = 11.8, 99% confidence interval = 10.0, 13.9, p<2.2e-16). Similarly in TG striatum, 91% of AcH3-associated genes also have a gene transcript present (Table 4; odds ratio = 7.3, 99% confidence interval = 6.2, 7.3, p<2.2e-16). Visualizing AcH3 association at a gene locus along with the presence of its transcripts along each chromosome highlights this association in both WT and TG striata (Figure 3). Here, the coincidence of histone H3 acetylation at the gene and the presence of the gene's transcript are easily seen (see insets in Figure 3). From this analysis, we conclude that the presence of acetylated histone H3 at a gene locus is sufficient to predict its expression and that this basic relationship between histone H3 acetylation and gene expression are unaffected in the R6/2 TG striatum.

**Table 3:** Histone H3 acetylation and gene expression in WT striata.

	Gene is expressed in WT (8655)	Gene is not expressed in WT (4164)
AcH3 present at gene in WT (4475 genes)	4171	304
AcH3 is not present at gene in WT (8344 genes)	4484	3860

**Table 4:** Histone H3 acetylation and gene expression in TG striata.

	Gene is expressed in TG (8582)	Gene is not expressed in TG (4237)
AcH3 present at gene in TG (3158 genes)	2883	275
AcH3 is not present at gene in TG (9661 genes)	5669	3962

### AcH3 localization within the gene region differs on gene transcription

**Figure 7:**
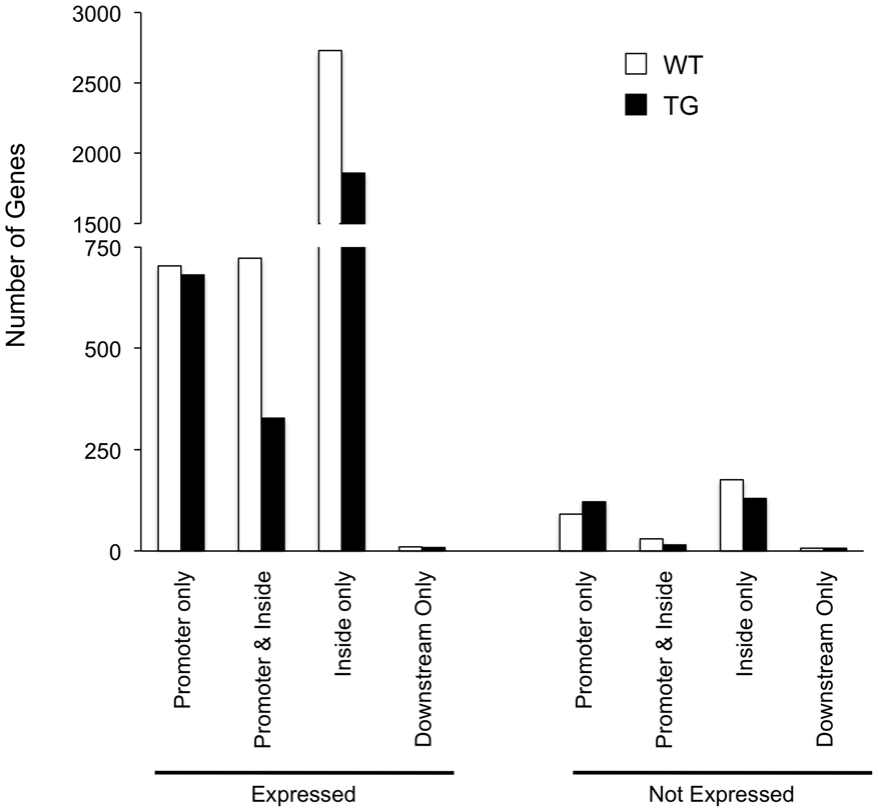
AcH3-localization within the gene changes upon gene expression. AcH3-localization within the gene changes on transcriptional status of the gene. White bars, WT; black bars, TG.

We furthered this analysis by examining the location of AcH3 bound probes within the gene locus itself. We divided genes by their expression pattern (expressed or not expressed) and then categorized them by the location of AcH3 binding (Figure 7). For expressed genes, we find the AcH3 is mostly associated at the inside coding regions and these numbers are decreased numbers in the TG striatum (Figure 7). A smaller number of AcH3-expressed gene associations occur at the promoter region and at both the promoter and inside regions, a pattern identical to the previous analysis that looked at all genes regardless of their transcriptional status. However, surprisingly, a similar number of genes were bound only at the promoter between WT and TG striata (Figure 7).

For genes that are not expressed, a similar trend was seen although the overall number of AcH3 bound genes was decreased. In this case, an unexpected pattern seen in the TG striatum was the increase over WT in the number of genes bound by AcH3 at the promoter (Figure 7). Of note, AcH3 binding appeared nearly equally distributed between binding at promoter and binding at inside regions for non-expressed genes.

### Differential histone H3 acetylation does not correlate well with differential gene expression

Throughout the course of our analysis, we noted that within our list of the top hypoacetylated genes, that the expression levels of five of these genes were downregulated—*Homer1*; *Cacna2d3*; *Sec14l1*; *Grik2* and *Rgs9*. To further investigate whether changes in histone H3 acetylation are associated with changes in gene expression between WT and TG striata, we examined which genes are differentially expressed and then interrogated their pattern of differential histone H3 acetylation. If we postulate that decreases in histone H3 acetylation correspond to decreases in the transcriptional activity of the associated gene, then we would expect to find that the majority of genes with decreased expression in the TG striatum also have decreases in histone H3 acetylation at the gene.

**Figure 8:**
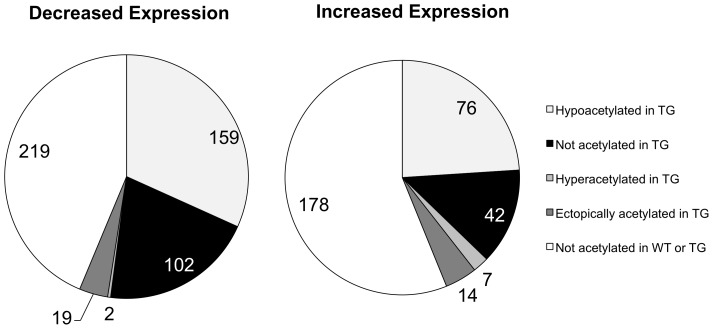
Gene expression changes are not strongly associated with changes in histone H3 acetylation in the R6/2 TG striatum. (A) Classification of differentially acetylated histone H3 genes whose expression are decreased in the TG striatum. (B) Classification of differentially acetylated histone H3 genes whose expression are increased in the TG striatum.

However, we find that the majority of genes with decreased expression in TG striata have no AcH3 associated at their genes in either WT or TG striata (Figure 8). Only 38% of downregulated genes have decreased levels of histone H3 acetylation at their gene loci. Unexpectedly, a similar pattern of differential histone H3 acetylation is also seen for genes with increased levels of expression in TG strata (Figure 8). Furthermore, an overwhelming majority of genes with both increases and decreases in expression levels do not have histone H3 acetylation association. Taken together, these data indicate that changes in histone H3 acetylation alone are not sufficient to account for changes in gene expression in the R6/2 HD model.

### HDAC inhibitor treatment does not act at specific gene loci

**Figure 9:**
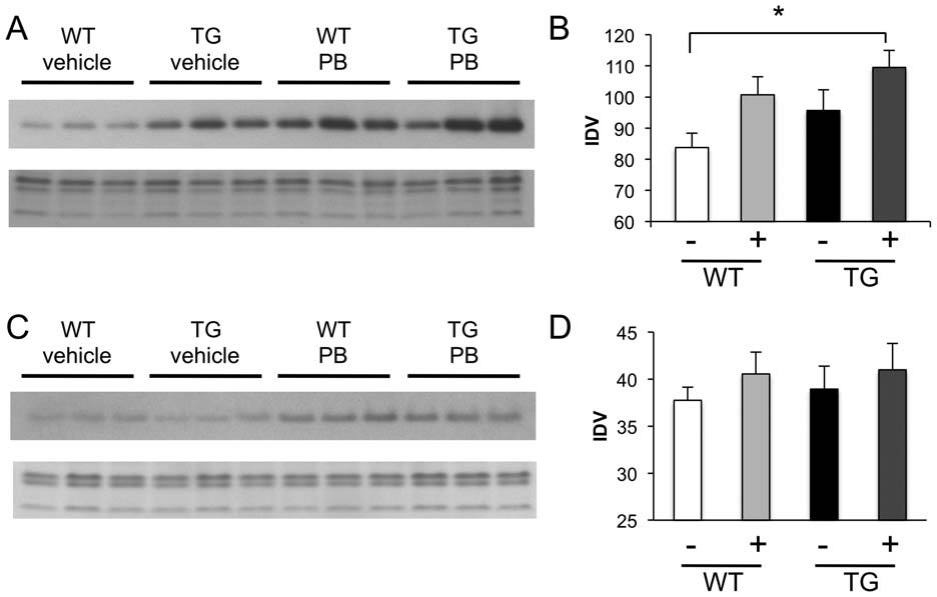
Phenylbutyrate treatment increases global histone H3 acetylation in R6/2 liver and cerebellum. (A) Western blot demonstrating AcH3 levels in histone extracts from WT and TG R6/2 livers (top), Coomassie blue gel staining demonstrates equal loading (bottom). (B) Integrated densitometry values (IDV) for AcH3 levels in A. (C) Western blot demonstrating AcH3 levels in histone extracts from WT and TG R6/2 cerebellum (top). Coomassie blue gel staining demonstrates equal loading (bottom). (D) Integrated densitometry values (IDV) for AcH3 levels in C. White bars, WT, vehicle (veh) treated (n = 9); Light grey bars, WT, phenylbutyrate (PB) treated (n = 8); Black bars, Tg, vehicle (veh) treated (n = 7); Dark grey bars, Tg, phenylbutyrate (PB) treated (n = 11). *, p-value<0.05. Error bars are standard deviation.

**Figure 10:**
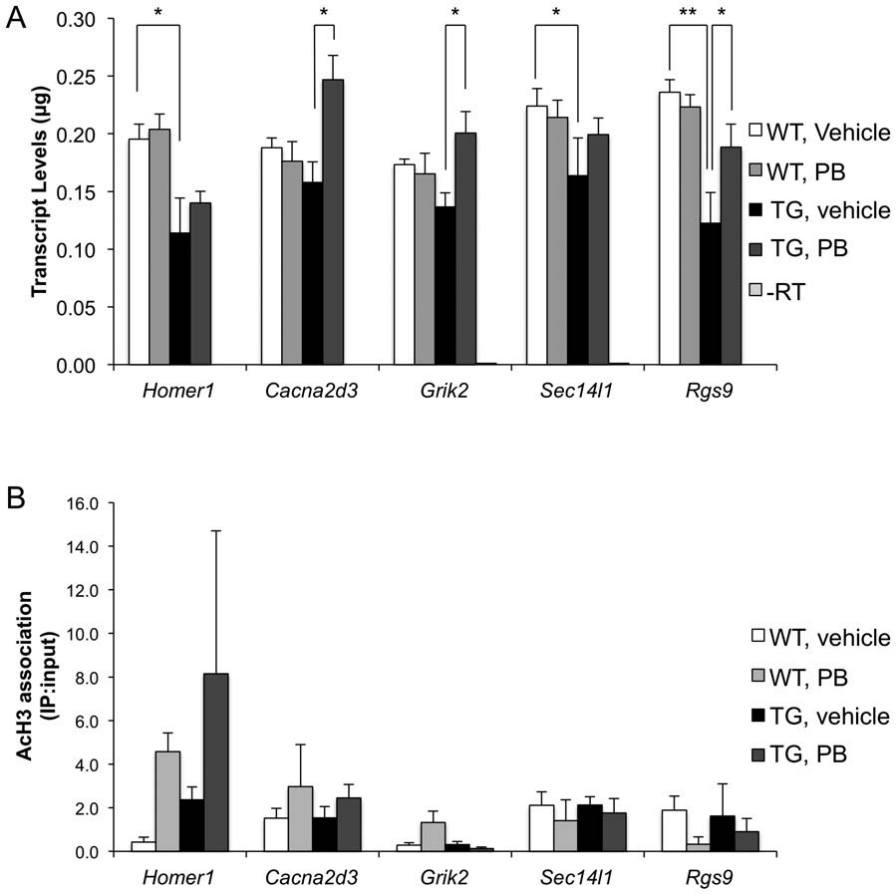
Phenylbutyrate treatment increases transcript levels of downregulated gene in TG striata but does not increase acetylation levels at gene loci. (A) Transcript levels of downregulated genes in the Tg striata. (B) Single-gene ChIP for the enrichment of AcH3 at gene regions. White bars, WT, vehicle treated; Light grey bars, WT, phenylbutyrate treated; Black bars, Tg, vehicle treated; Dark grey bars, Tg, phenylbutyrate treated. *, p<0.05; **, p<0.01. Error bars are standard deviation.

Another surprising result from our study reveals that global levels of AcH3 do not predict the degree of genomic binding of AcH3. In fact, globally, AcH3 levels are marginally increased in the livers and cerebella of TG mice, as compared to WT littermates (Figure 9). Therefore, in order to determine if global HDAC inhibitor treatment changes histone H3 acetylation at individual gene loci, we treated 12-week R6/2 TG and WT littermates with phenylbutyrate or vehicle and examined changes in global histone H3 acetylation levels, gene expression and histone H3 acetylation at specfic gene loci. As expected, we find that phenylbutyrate treatment increases global histone H3 acetylation in the histone extracts from WT and TG liver (Figure 9A, B) and cerebella (Figure 9C, D). At the same time, transcript levels of downregulated genes are increased in TG striata following phenylbutyrate treatment (Figure 10A). However, at specific gene loci, only two, *Homer1* and *Cacna2d3*, of the five genes tested have increased histone H3 acetylation in the TG striatum following phenylbutyrate treatment (Figure 10B). These results suggest that changes in the global chromatin environment rather than at specific gene loci may be more important for causing transcriptional changes as a result of HDAC inhibitor treatment.

## Discussion

### Genome-wide histone H3 acetylation in the normal and HD mammalian striatum

This study is the first to analyze histone H3 K9/K14 acetylation (AcH3) at gene loci and gene expression across the entire genome in the mammalian brain. In the R6/2 mouse model for Huntington's disease (HD) and the corresponding control littermates, we analyzed genome-wide histone H3 acetylation at gene loci. An important point to note from our study is that global levels of acetylated histone H3 do not predict its levels at the genome. Though global levels were similar if not slightly elevated in TG animals, overall histone H3 K9/K14 acetylation at gene loci was decreased in the TG striatum. Furthermore, we observed in both WT and TG striatum that AcH3 localization within the gene locus was unevenly distributed and was more likely to occur within the coding region—downstream of the transcriptional start site rather than the region of the core promoter which is upstream of the transcriptional start site.

### Histone H3 acetylation at gene loci predicts gene expression in WT and TG striata

Another novel result from our study comes with correlating AcH3 at gene loci with gene expression data. It is well known that increases in histone H3 acetylation precede increases in transcriptional activity [10]–[13] and similar correlations between histone H3 acetylation and gene expression have been made at the single gene level in multiple studies of human cell lines [19]–[23]. Our data are the first to demonstrate this important relationship in a normal mammalian brain as well as in a transgenic model for HD. We found a strong correlation between the presence of acetylated K9/K14 histone H3 at a gene locus and the presence of the gene's transcript. While this finding demonstrates that histone acetylation is sufficient for gene transcription, our data do not support the notion that it is also necessary as a large number of expressed genes do not have acetylated histone H3 present at their gene locus.

### Differential histone H3 acetylation does not predict transcriptional changes in HD

Furthermore, we did not find a clear pattern between differential changes in histone H3 K9/K14 acetylation with changes in gene expression between WT and TG striata. Therefore, as changes in histone H3 acetylation alone are not adequate to explain changes in gene expression, some additional mechanism must account for the downstream transcriptional changes seen in HD. This may not be too surprising given that we find fewer than half of expressed genes in the normal WT brain (and even fewer in the TG, see Tables 3 and 4) actually have associated AcH3 at their loci. These data together indicate that while histone H3 acetylation at specific gene loci is sufficient to cause increases in gene expression, there may be other mechanisms at play requiring other factors or histone modifications. One such factor may be Huntingtin (Htt) itself. Htt interactions with genomic DNA could influence transcription as we have demonstrated that Htt can interact with genomic DNA *in vivo* and *in vitro* with both wild-type and mutant forms of Htt interacting at different locations within the genome [24].

### Mechanisms of systemic HDAC inhibitor treatments?

Results from our study suggest that global levels of histone H3 K9/K14 acetylation levels do not predict the levels of AcH3 present at chromatin. And despite increased global acetylated histone H3 levels and increased transcript levels of downregulated genes following HDAC inhibitor treatments, AcH3 levels at the gene were not significantly altered.

However, broad-spectrum HDAC inhibitors clearly have beneficial effects in animal models of various neurodegenerative diseases and dramatically improve multiple phenotypes. Broad-spectrum HDAC inhibitors, such as sodium butyrate, result in increased global levels of acetylated histone H3 and H4 [25]–[28]. Furthermore, HDAC inhibitors improve molecular, cellular and behavioral phenotypes in animal models for a number of neurodegenerative diseases including HD [6], [29]–[34], Parkinson's disease [35], spinal and bulbar muscular atrophy [36], Alzheimer's disease [37], [38], dentatorubral-pallidoluysian atrophy [39], amyotrophic lateral sclerosis [40], [41] and Friedreich's ataxia [42].

If their effects are not directly on histone acetylation, how could they convey such a beneficial action? Acetylation is a relatively transient form of histone modification and may not correlate well with steady-state mRNA measurements. Thus, simply reversing decreases in AcH3 by HDAC inhibitor treatment in the TG striatum may have a beneficial impact on overall chromatin structure and the epigenetic environment. Such increases in overall acetylation levels by HDAC inhibitor treatment could then lead to changes of other histone modifications, such as ubiquitylation or methylation. Such trans-histone crosstalk is well documented for activating as well as repressive histone modifications, is predicted by the histone code [10], [43] and has the potential to consolidate a transient modification such as acetylation into more stable, long-lived modifications such as ubiquitylation or methylation. For example, crosstalk between acetylation and phosphorylation plays a role in transcriptional elongation [44]. Coordination of acetylation on histone H3 and trimethylation of lysine residue 4 of histone H3 (H3K4me3), both of which are associated with active transcription, occurs through subunits of histone acetylatransferase complexes [45], [46] allowing for binding of TFIID of the RNA polymerase complex to H3K4me3 [47]. Furthermore, HDAC inhibitor treatment of human cell lines increases histone acetylation with concomitant decreases in DNA methylation, a well-known indicator of silenced genes [48].

Additionally, HDAC inhibitor therapy may also exert effects on the non-transcriptional and nuclear, non-histone targets of HDACs as well. Disrupting HDAC1 and HDAC2 function alters their interactions with members of the Wnt signaling pathway [49]. HDACs also target non-nuclear proteins such as alpha-tubulin [50], the acetylation of which is thought to play a role in HD progression [51]. Mutant forms of Htt (mHtt) can themselves be acetylated which facilitates their clearance from the cell while inhibiting HDAC1 increases acetylated forms of mHtt and improved mHtt clearance from the cell [52].

In summary, this is the first study to demonstrate a strong link between histone acetylation and gene expression in the normal brain as well as an HD model. Global levels of AcH3 are not predictive of the degree of genomic binding of AcH3. We find that histone H3 acetylation strongly correlates with gene expression in both WT and TG R6/2 striata. AcH3 levels at gene loci are decreased in the TG striatum; however, these decreases do not correlate with changes in transcript levels of individual genes in HD. These observations suggest that gene expression changes may be related to the overall chromatin environment, part of which is determined by histone H3 K9/K14 acetylation. Furthermore, our data reveal that the mechanistic relationship between histone H3 K9/K14 acetylation at gene loci and active gene expression is unchanged in an HD model, shedding light onto other potential mechanisms for the action of HDAC inhibitor therapy in the treatment of HD and other neurodegenerative disorders.

## Materials and Methods

### Animals

#### Ethics Statement

All animal care, husbandry and experimentation were performed with the approval of and according to the guidelines set by the Massachusetts General Hospital Subcommittee on Research Animal Care.

#### Mouse Husbandry & Tissue Procurement

Transgenic (TG) and wild-type (WT) littermate controls from the R6/2 mouse line were used for these experiments. The TG R6/2 mouse contains the exon 1 portion of the human *Huntingtin* gene containing the CAG repeat expanded to 110 to 150 repeats and is expressed under the control of the human *Huntingtin* promoter [16]. The R6/2 line was maintained by backcrossing genotyped R6/2 TG males to F1 hybrid females from a C57BL/6 male x CBA female cross (B6CBAF1/J; The Jackson Laboratory, Bar Harbor, ME) as described [16]. In our R6/2 colony, the CAG repeat size expanded to approximately 200 repeats. 12-week old R6/2 wild-type animals were sacrificed by the administration of carbon dioxide and their brains were quickly removed and regionally dissected over ice before snap-freezing in liquid isopentane cooled with dry ice. Brains were stored at −80°C until needed.

#### Phenyl Butryate treatment

Twelve week old R6/2 TG and WT littermates were injected with phenylbutyrate daily by intraperitoneal injection (400 mg/kg/day, SPB11, Scandinavian Formula, Sellersville, PA) or vehicle (phosphate buffered saline) for seven consecutive days and sacrificed on the eighth day as described above. Each treatment group contained a minimum of six animals. Livers and cerebella were dissected to determine global acetylated histone H3 levels while the striata were used for ChIP assays. Analyses were performed on the dissected tissue from each animal individually.

### Chromatin immunoprecipitation (ChIP) assay

ChIP homogenates were prepared from the dissected striata of R6/2 TG and WT animals according to published protocols [53], [54]. For ChIP-chip experiments, each biological replicate contained striata from 8 animals (16 striata total). Each experiment contained two biological replicates. For single gene, confirmatory ChIP, a total of 4–6 biological replicates were used. Protein-DNA interactions were captured by formaldehyde crosslinking and genomic DNA was sheared to lengths of ∼500 bp using a probe sonicator (Fisher Scientific, Pittsburgh, PA, Sonic Dismembrenator Model 500). Prior to ChIP, a sample of each homogenate representing 1% of the total volume was removed and set aside as input, i.e. whole-cell extract (WCE). Antibodies directed against di-acetylated histone H3 on K9 and K14 (AcH3; Millipore, Billerica, MA; catalog number 06-599) were used in ChIP reactions. Negative controls included IgG (Jackson ImmunoResearch, West Grove, PA) or no antibody conditions.

IP and WCE products from ChIP were quantified using the luciferase- and T4 DNA polymerase-based DNA Quantitation System (Promega, Madison, WI) according to manufacturer's instructions. The light output was immediately measured in a luminometer (TD-20/20 Luminometer, Turner Designs, Sunnyvale, CA). ChIP products were also quantified using Quant-iT PicoGreen dsDNA reagent (Invitrogen, Carlsbad, CA) and assayed on the Wallac Victor 1420 Multilabel Counter (PerkinElmer, Waltham, MA) with excitation and emission wavelengths of 485 nm and 535 nm, respectively.

### ChIP-chip

#### Whole Genomic Amplification

Recovered IP and WCE DNA products were amplified using the GenomePlex Whole Genome Amplification kit (Sigma, St. Louis, MO). Ten ng of WCE or ChIP DNA was used in each reaction and the manufacturer's procotol was followed with the exception of skipping the initial random fragmentation step. The resulting product was purified using the QIAquick-PCR purification kit (Qiagen, Valencia, CA) and DNA yields were measured using a NanoDrop (ThermoScientific, Waltham, MA).

#### Labeling

IP and WCE amplified products were fluorescently labeled with Cy5- and Cy3-dUTP (GE Healthcare, Piscataway, NJ), respectively, using the Klenow-based BioPrime Array CGH Genomic Labeling kit (Invitrogen). 2 ug of amplified DNA was used in each reaction. Unincorporated fluorescent nucleotides were removed from DNA products using the purification columns included in the kit. Labeled products were quantified on a NanoDrop (ThermoScientific).

#### Hybridization and Washing

Amplified and labeled IP (Cy5) and WCE (Cy3) DNA products were hybridized to Agilent Technologies (Santa Clara, CA) mouse promoter arrays (G4490A; UCSC mm7/NCBI release 35, August 2005). These promoter arrays cover approximately 18,000 mouse transcripts with an average of 25 probes per gene. The 60-mer oligonucleotide probes are spaced every ∼100–300 bp and cover −5.5 kb to +2.5 kb for a typical gene (transcriptional start site is denoted as +1).

IP and WCE-labeled DNA products (5 ug each) were combined and co-hybridized in hybridization buffer in the presence of blocking agent and mouse cot-1 DNA at a final concentration of 0.1 mg/ml. All hybridization, washing and drying buffers as well as the blocking agent were supplied as part of the Agilent aCGH hybridization kit. Arrays were placed in the Agilent hybridization chambers and hybridized in a rotisserie hybridization oven set at 20 rpm for 40 h at 65°C. Arrays were washed as follows: hybridization chamber disassembly in Oligo aCGH Wash Buffer 1 at room temperature, Oligo aCGH Wash Buffer 1 at room temperature for 5 min, Oligo aCGH Wash Buffer 2 at 37°C for 1 min, acetonitrile wash at room temp for 1 min and a final incubation in the Stabilization and Drying Solution (Agilent) for 1 min. Microarray slides were scanned at a 5 µm resolution using the Agilent scanner. Raw fluorescence intensities were obtained from scanned images using Agilent's Feature Extraction Software. Background and noise were removed from the data and the feature extractions were monitored. Fluorescent intensities of Cy5/Cy3 (IP/WCE ratios) were calculated for each individual feature on the array and represent the binding events of the amplified ChIP product.

#### Data Analysis

Pre-processed data were normalized and analyzed using the ChIP Analytics Software 1.3 (Agilent). Replicates were normalized together using the intra-array LOWESS intensity dependent normalization and the results were averaged using the replicate support extended Whitehead error model v1.0 and the peak detection and evaluation was computed using the Whitehead per-array Neighbourhood Model v1.0. Results of the combined analyses were used to generate spreadsheet reports for the probes, segments and gene levels results, UCSC track files, Ensembl DAS files and quality control reports. Probe intensity values were calculated by:


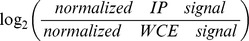


For qualitative analyses, we analyzed the ChIP-chip data at the probe and gene levels in a binary bound/not bound fashion. An individual probe was defined as bound if p≤0.005, while a gene was defined as bound if at least one probe representing that gene is bound.

For quantitative analyses, we determined differentially bound genes between WT and TG samples by calculating a composite score for each gene by subtracting the summation of all WT bound probes from the summation of all TG bound probes as follows:


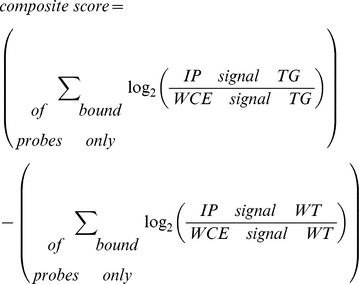


#### Gene Expression Analysis

Using a previously published gene expression microarray dataset [17] from 12-week old R6/2 TG and WT littermates, we used Affymetrix MAS5 analysis to normalize probe intensities and monitor present/absent calls. Since a single gene can be represented across multiple probesets, we calculated the expression value for each gene as the mean for all probesets representative for that gene. We defined genes as expressed in WT or TG striata when the average expression value was greater than 100. To determine differentially expressed genes, tests were computed with two-tailed unpaired t-test for unknown variance and unpaired two classes analysis using the Significance Analysis of Microarrays (SAM) algorithm using Bioconductor and R [55]. We defined genes as up- or down-regulated when the differential expression ratio was at least 1.5-fold and the difference between the two groups was statistically significant (p≤0.05).

#### Combined Gene Expression and ChIP-chip datasets

Since the ChIP-chip (containing >18,000 genes) and Affymetrix gene expression (containing >20,000 genes) microarray datasets do not contain the same set of genes, we defined the common gene set present on both microarrays as the union of each individual microarray gene list. Within this common list, information for each gene was extracted from the microarray dataset as well as the ChIP-chip dataset and were combined into a common spreadsheet. This combined ChIP-chip/expression dataset was used for further analysis using Microsoft Excel.

#### GO functional annotation

Gene lists for “Hypoacetylated in TG,” “Not acetylated in TG,” “Hyperacetylated in TG,” and “Ectopically acetylated in TG” categories were analyzed using DAVID 6.7 (http://david.abcc.ncifcrf.gov/home.jsp) for functional annotation clustering [18]. Annotation clusters with enrichment scores greater than 1.3 are reported.

### Primer design for single-gene ChIP and RT-qPCR validation assays

To determine differentially acetylated genes, we ranked the genes by composite score. Gene-specific primers for single-gene ChIP were designed based on differentially bound probes. Using the genomic location of differentially bound probes based on the annotation provided by Agilent, sequences from each region were obtained from the UCSC mm7 browser (http://genome.ucsc.edu/cgi-bin/hgGateway?db=mm7) and used to design gene-specific primers for single-gene ChIP. Gene-specific primers for RT-qPCR were designed to the region containing the 5′UTR and the start codon as described by the annotation provided in NCBI's accession record for the mRNA of each gene (found at http://www.ncbi.nlm.nih.gov/nuccore/). Primer-BLAST [56] at the NCBI website (http://www.ncbi.nlm.nih.gov/tools/primer-blast/index.cgi?LINK_LOC=BlastHome) was used to design primers. Prior to their use in qPCR reactions, primers were tested in a conventional PCR reaction using genomic DNA as template and checked on an agarose gel to ensure the primers resulted in a single band of expected size. Primer sequences for single-gene ChIP and RT-qPCR are provided in Tables S1 and S2, respectively.

### Single-gene ChIP assays

Input and IP samples were interrogated with gene-specific primers in replicate reactions in real-time PCR analysis as previously described [53], [54]. Using iCycler software (BioRad, Hercules, CA), threshold amplification cycle numbers (C_T_) were used to calculate IP DNA quantities as ratios of corresponding inputs. IP to input ratios of AcH3-gene associations were calculated as follows: log_2_ (C_t_ of IP/C_t_ of input).

### Histone extractions

Cerebella and livers from phenylbutyrate- and vehicle-treated R6/2 TG and WT littermates were used. In order to minimize the number of mice for these experiments, cerebellar tissues were used as “in-brain” controls in order to assess the efficacy of phenyl butyrate treatments, reserving the striatal tissue for ChIP experiments. Histones were acid-extracted as follows. Tissues were homogenized in ice-cold 5% Triton buffer (5% Triton, 3 mM DTT, 1 mM sodium orthovanadate, 5 mM sodium fluoride, 1 mM PMSF, 5 mM sodium butyrate in 1× PBS containing a protease inhibitor cocktail). Cells were lysed on ice and nuclei were collected by gentle centrifugation. Nuclei were washed 3× in ice cold 5% Triton buffer and proteins were extracted in 0.2 M HCl with vigorous shaking at 4°C for 3 hrs. Supernatants were cleared by centrifugation and neutralized with 1 M NaOH to a neutral pH. Protein concentrations were determined using the Bradford reagent (BioRad).

### Western blots

Histone extracts were diluted in 2× Tricine SDS sample buffer (Invitrogen) and boiled for 5 min. Proteins were fractionated on a 10–20% Tricine gel (Invitrogen) at 120 V until the dye front reached the bottom of the gel. Proteins were then transferred to PVDF using the iBlot transfer system (Invitrogen) according to the manufacturer's instructions. Coomassie blue staining of the gels after transfer was used to ensure equal loading across wells. Membranes were blocked in 5% non-fat dry milk in TBST (50 mM Tris, 150 mM NaCl, 0.1% Tween 20) for 1 h at room temperature before incubation with primary antibodies. Following primary antibody (AcH3; 05-699, Millipore) incubation, blots were washed in TBST and then incubated in secondary antibody of HRP-conjugated goat anti-rabbit IgG (Jackson ImmunoResearch). Immunocomplexes were visualized with the Western Lightning Plus enhanced chemiluminescent (ECL) detection system (PerkinElmer) and exposed to film (GE Healthcare).

### RNA extractions and RT-qPCR

Total RNA was extracted from a single striatum of phenylbutyrate- or vehicle-treated R6/2 TG and WT littermates using the RNeasy Mini kit (Qiagen) following the manufacturer's instructions. Total RNA was quantified using a spectrophotometer and cDNA was generated using equal amounts of total RNA, random hexamers and the SuperScript First Strand reaction kit (Invitrogen). Transcript levels were quantified as described [7]. Briefly, cDNA was used as template with gene specific primers in a PCR reaction using the SYBR Green PCR Master Mix (Applied Biosystems, Foster City, CA). Real-time thermal cycling was performed using an iCycler thermal cycler (Bio-Rad, Hercules, CA), with continuous SYBR Green monitoring according to the manufacturer's specifications.

## Supporting Information

Table S1 **Gene-specific primer sequences for single-gene ChIP confirmation experiments.**
(DOCX)

Table S2 **Gene-specific primer sequences for RT-qPCR analysis.**
(DOCX)

Table S3 **Gene Ontology (GO)-Biological Process (GOTERM_BP_FAT) Functional Annotation Clustering of “Hypoacetylated in TG” genes.**
(DOCX)

Table S4 **Gene Ontology (GO)-Biological Process (GOTERM_BP_FAT) Functional Annotation Clustering of “Not acetylated in TG” genes.**
(DOCX)

Table S5 **Gene Ontology (GO)-Biological Process(GOTERM_BP_FAT) Functional Annotation Clustering of “Hyperacetylated in TG” genes.**
(DOCX)

Table S6 **Gene Ontology (GO)-Biological Process (GOTERM_BP_FAT) Functional Annotation Clustering of “Ectopically acetylated in TG” genes.**
(DOCX)
