# Supplementary material for: Genome-Wide Histone Acetylation Is Altered in a Transgenic Mouse Model of Huntington's Disease
Source: PLoS One. 2012 Jul 27;7(7):e41423. doi: 10.1371/journal.pone.0041423 (PMC3407195; doi:10.1371/journal.pone.0041423)
Supplement: Table S1 — Gene-specific primer sequences for single-gene ChIP confirmation experiments. (DOCX) [file pone.0041423.s001.docx]

**Supplemental Table 1**: Gene-specific primer sequences for single-gene ChIP confirmation experiments.

| **Ranking** | **Gene Primer** | **Sequence** | **Genomic location (bp)** |
| --- | --- | --- | --- |
| **Decreased acetylation in TG** | | | |
| **1** | ***Zfp469*** |  | Chr 12 |
|  | F1304 | ACCTTTTTAGGGCCCTCGTCTCCC | 141850304 |
|  | R1435 | AACCCGTTTCGTATCTGACCTTGGTA | 141850435 |
| **2** | ***Per1*** |  | Chr 11 |
|  | F1575 | TGTCTAGACGTTCGGTCGGGCA | 69177475 |
|  | R1674 | AACCCGAGATCACTCGAGAAGCC | 69177574 |
| **3** | ***BC057627/Zc3h4*** |  | Chr 7 |
|  | F5029 | ACCGTTGCAAGCAGGCCCAT | 14165229 |
|  | R5177 | AGTGGACAGGAGAGATCAGGGCT | 14165377 |
| **4** | ***Pik3r1*** |  | Chr 13 |
|  | F3231 | GCCCCTGGGAGCCAGAACCT | 98802031 |
|  | R3376 | CCCCCACCCCCGTGTTGTGTA | 98802176 |
| **5** | ***Homer1*** |  | Chr 13 |
|  | F6753 | GCTGGGAACATGTGTTATGGAGTGG | 90368953 |
|  | R6867 | TGCCAACCTTGTTCCTCAACTACT | 90369067 |
| **6** | ***Lrrtm2*** |  | Chr 18 |
|  | F3418 | GTGAGAGCTAGTGTTAGACTGCCG | 35591418 |
|  | R3531 | ACCAGAGCAGAGGCGTACCT | 35591531 |
| **9** | ***Chn1*** |  | Chr 2 |
|  | F1895 | AGAGCACAGGGAGGTGCAGG | 73503795 |
|  | R2041 | GGTAGCACAGACTGATTTCCTCTGC | 73503941 |
| **30** | ***Rgs9*** |  | Chr 11 |
|  | F1846 | TGCAGCAGACCATCACGGCT | 109406746 |
|  | R1976 | CGATCCGCGCTGCCCAATTT | 109406876 |
| **123** | ***Gsk3b*** |  | Chr 16 |
|  | F7311 | TGCCGAGTCCTTGTCTTCTGGA | 37797811 |
|  | R7446 | ACTTTGTGTAGTCACACGGGCA | 37797946 |
| **Increased acetylation in TG** | | | |
| **1** | ***Zfp367*** |  | Chr 13 |
|  | F18909 | TCCCGGTTCCTTCCGAGCAC | 61975409 |
|  | R19024 | ACATCCGGAGCAGCTGAGGG | 61975524 |
| **2** | ***A430060F13Rik*** |  | Chr 11 |
|  | F2802 | AGCATTGAGCTTGACGCGTGGT | 39460902 |
|  | R2916 | GGTCACGTTGAAAGGCCTCTATGC | 39461016 |
| **4** | ***Xrn1*** |  | Chr 9 |
|  | F3594 | CCTCCTAAGTAACCACAACCGGC | 95599294 |
|  | F3704 | CCCACCATTTCAAGACTCTGGGT | 95599404 |
| **5** | ***Slc25a37*** |  | Chr 14 |
|  | F2696 | TGAAGCGGAGTGGTTGGACA | 64147996 |
|  | R2799 | TTCCCTGGAATGGGCCCTAGC | 64148099 |
| **6** | ***Fgf9*** |  | Chr 14 |
|  | F4127 | ACCTGCAATATTGTTTGCGCGTC | 52939727 |
|  | R4266 | TTCGCGCGGACTCTGTAACC | 52939866 |
| **7** | ***Chuk*** |  | Chr 19 |
|  | F525 | GCTGAAGCAAAGGGGTACATGC | 43968825 |
|  | R626 | CCTGTGTGGTAGCATTGTGTCG | 43968926 |
| **8** | ***Ptn*** |  | Chr 6 |
|  | F2360 | AAGCAGGCTACTCTGGGGGC | 36741860 |
|  | R2459 | ATATGGTGCTGGGTGGGTGCT | 36741959 |
| **9** | ***Cwf19l2*** |  | Chr 9 |
|  | F4769 | GTCAGGCAAGAAGGGGGAAGT | 3314903 |
|  | R4946 | GCGGAAACTTGTAGCCTGCTG | 3315808 |
| **10** | ***Klhl7*** |  | Chr 5 |
|  | F6252 | AAGGCCAAACACTGGTCGCA | 23142152 |
|  | R6393 | AGAAGCCAGCCATTCACAGC | 23142293 |
