# Supplementary material for: Genome-Wide Histone Acetylation Is Altered in a Transgenic Mouse Model of Huntington's Disease
Source: PLoS One. 2012 Jul 27;7(7):e41423. doi: 10.1371/journal.pone.0041423 (PMC3407195; doi:10.1371/journal.pone.0041423)
Supplement: Table S2 — Gene-specific primer sequences for RT-qPCR analysis. (DOCX) [file pone.0041423.s002.docx]

**Supplemental Table 2:** Gene-specific primer sequences for RT-qPCR.

| ***homer homolog 1 (Drosophila)*** | | ***Homer1*** |
| --- | --- | --- |
|  | NM_147176.2 | translational start site @ nt 804 |
| U852 | GACCCGAACACAAAGAAGAACTGGGTA |  |
| L1049 | TGCCCGGCTATCAGCCCATT |  |
| ***glutamate receptor, ionotropic, kainate 2 (beta 2)*** | | ***Grik2, aka Glur6*** |
|  | NM_010349.2 | translational start site @ nt 414 |
| U372 | GATCGGGGAAGTGGGTGCCG |  |
| L568 | GCTCCCATAGGGCCAGATTCCA |  |
| ***SEC14-like 2 (S. cerevisiae)*** | | ***Sec14l2*** |
|  | NM_028777.3 | translational start site @ nt 211 |
| U223 | TACCAGTCCCCAGTGCGGGT |  |
| L395 | TCCAGCTTGCAGCGCCTCTC |  |
| ***calcium channel, voltage-dependent, alpha2/delta subunit 3*** | | ***Cacna2d3*** |
|  | NM_009785.1 | translational start site @ nt 98 |
| U230 | TGGGCCTCCGCTTTTGGTGG |  |
| L421 | ACGCCTCACTGCCTCGGACT |  |
| ***regulator of G-protein signaling 9*** | | ***Rgs9*** |
|  | NM_011268.2 | translational start site @ nt 188 |
| U205 | AGGCCAGCAGTACAGGCCGA |  |
| L334 | GGCGTGAGGGACACTGGTGA |  |

**Abbreviations**

AcH3, acetylated histone H3

ChIP, Chromatin ImmunoPrecipitation

ChIP-chip, Chromatin Immunopreciptation coupled with microarray hybridization

HD, Huntington’s disease

HDAC, histone deacetylase enzyme

TG, transgenic R6/2 mice

WCE, whole cell extract

WT, wild-type R6/2 mice
