# Supplementary material for: Genome-Wide Histone Acetylation Is Altered in a Transgenic Mouse Model of Huntington's Disease
Source: PLoS One. 2012 Jul 27;7(7):e41423. doi: 10.1371/journal.pone.0041423 (PMC3407195; doi:10.1371/journal.pone.0041423)
Supplement: Table S3 — Gene Ontology (GO)-Biological Process (GOTERM_BP_FAT) Functional Annotation Clustering of “Hypoacetylated in TG” genes. (DOCX) [file pone.0041423.s003.docx]

Supplemental Table 3: Gene Ontology (GO)-Biological Process (GOTERM_BP_FAT) Functional Annotation Clustering of “Hypoacetylated in TG” genes

| **Term** | **Count** | **%** | **PValue** | **List Total** | **Pop Hits** | **Pop Total** | **Fold Enrichment** | **Bonferroni** | **Benjamini** | **FDR** |
| --- | --- | --- | --- | --- | --- | --- | --- | --- | --- | --- |
| **Annotation Cluster 1**  **Enrichment Score: 11.99027672516445** |  |  |  |  |  |  |  |  |  |  |
| GO:0046907~intracellular transport | 124 | 5.03 | 1.33E-17 | 1810 | 431 | 13588 | 2.16 | 4.53E-14 | 4.53E-14 | 2.43E-14 |
| GO:0045184~establishment of protein localization | 160 | 6.49 | 6.01E-15 | 1810 | 656 | 13588 | 1.83 | 2.04E-11 | 4.08E-12 | 1.09E-11 |
| GO:0015031~protein transport | 158 | 6.40 | 1.47E-14 | 1810 | 651 | 13588 | 1.82 | 4.99E-11 | 8.32E-12 | 2.68E-11 |
| GO:0008104~protein localization | 174 | 7.05 | 6.79E-14 | 1810 | 753 | 13588 | 1.73 | 2.31E-10 | 2.57E-11 | 1.24E-10 |
| GO:0006886~intracellular protein transport | 75 | 3.04 | 1.31E-09 | 1810 | 276 | 13588 | 2.04 | 4.45E-06 | 2.62E-07 | 2.38E-06 |
| GO:0070727~cellular macromolecule localization | 79 | 3.20 | 2.56E-09 | 1810 | 301 | 13588 | 1.97 | 8.73E-06 | 4.60E-07 | 4.68E-06 |
| GO:0034613~cellular protein localization | 78 | 3.16 | 4.38E-09 | 1810 | 299 | 13588 | 1.96 | 1.49E-05 | 7.47E-07 | 8.01E-06 |
| **Annotation Cluster 2**  **Enrichment Score: 11.550893562326817** |  |  |  |  |  |  |  |  |  |  |
| GO:0006396~RNA processing | 123 | 4.99 | 2.25E-16 | 1810 | 437 | 13588 | 2.11 | 7.56E-13 | 3.78E-13 | 4.11E-13 |
| GO:0016071~mRNA metabolic process | 87 | 3.53 | 1.80E-12 | 1810 | 302 | 13588 | 2.16 | 6.11E-09 | 5.10E-10 | 3.28E-09 |
| GO:0006397~mRNA processing | 75 | 3.04 | 9.83E-11 | 1810 | 262 | 13588 | 2.15 | 3.35E-07 | 2.23E-08 | 1.79E-07 |
| GO:0008380~RNA splicing | 60 | 2.43 | 1.57E-09 | 1810 | 201 | 13588 | 2.24 | 5.36E-06 | 2.98E-07 | 2.88E-06 |
| **Annotation Cluster 3**  **Enrichment Score: 11.224716723994085** |  |  |  |  |  |  |  |  |  |  |
| GO:0044265~cellular macromolecule catabolic process | 153 | 6.20 | 1.77E-15 | 1810 | 609 | 13588 | 1.89 | 6.05E-12 | 2.02E-12 | 3.24E-12 |
| GO:0009057~macromolecule catabolic process | 161 | 6.53 | 2.03E-15 | 1810 | 654 | 13588 | 1.85 | 6.81E-12 | 1.70E-12 | 3.65E-12 |
| GO:0030163~protein catabolic process | 139 | 5.63 | 6.06E-14 | 1810 | 556 | 13588 | 1.88 | 2.06E-10 | 2.58E-11 | 1.11E-10 |
| GO:0051603~proteolysis involved in cellular protein catabolic process | 133 | 5.39 | 3.06E-13 | 1810 | 534 | 13588 | 1.87 | 1.04E-09 | 1.04E-10 | 5.59E-10 |
| GO:0044257~cellular protein catabolic process | 133 | 5.39 | 4.75E-13 | 1810 | 537 | 13588 | 1.86 | 1.62E-09 | 1.47E-10 | 8.68E-10 |
| GO:0043632~modification-dependent macromolecule catabolic process | 121 | 4.90 | 9.19E-11 | 1810 | 508 | 13588 | 1.79 | 3.13E-07 | 2.24E-08 | 1.68E-07 |
| GO:0019941~modification-dependent protein catabolic process | 121 | 4.90 | 9.19E-11 | 1810 | 508 | 13588 | 1.79 | 3.13E-07 | 2.24E-08 | 1.68E-07 |
| GO:0006508~proteolysis | 166 | 6.73 | 5.97E-03 | 1810 | 1034 | 13588 | 1.21 | 1.00E+00 | 1.65E-01 | 1.04E+01 |
| **Annotation Cluster 4**  **Enrichment Score: 6.631693686523574** |  |  |  |  |  |  |  |  |  |  |
| GO:0033554~cellular response to stress | 100 | 4.05 | 5.22E-10 | 1810 | 404 | 13588 | 1.86 | 1.78E-06 | 1.11E-07 | 9.54E-07 |
| GO:0006281~DNA repair | 59 | 2.39 | 2.08E-07 | 1810 | 222 | 13588 | 2.00 | 7.07E-04 | 3.07E-05 | 3.79E-04 |
| GO:0006974~response to DNA damage stimulus | 71 | 2.88 | 2.21E-07 | 1810 | 287 | 13588 | 1.86 | 7.51E-04 | 3.13E-05 | 4.03E-04 |
| GO:0006259~DNA metabolic process | 84 | 3.40 | 1.24E-04 | 1810 | 421 | 13588 | 1.50 | 3.45E-01 | 8.43E-03 | 2.27E-01 |
| **Annotation Cluster 5**  **Enrichment Score: 6.595296922815506** |  |  |  |  |  |  |  |  |  |  |
| GO:0032446~protein modification by small protein conjugation | 31 | 1.26 | 3.16E-08 | 1810 | 79 | 13588 | 2.95 | 1.08E-04 | 5.12E-06 | 5.76E-05 |
| GO:0070647~protein modification by small protein conjugation or removal | 35 | 1.42 | 2.80E-07 | 1810 | 104 | 13588 | 2.53 | 9.53E-04 | 3.53E-05 | 5.11E-04 |
| GO:0016567~protein ubiquitination | 25 | 1.01 | 1.85E-06 | 1810 | 66 | 13588 | 2.84 | 6.29E-03 | 2.10E-04 | 3.38E-03 |
| **Annotation Cluster 6**  **Enrichment Score: 4.776700237783898** |  |  |  |  |  |  |  |  |  |  |
| GO:0022613~ribonucleoprotein complex biogenesis | 40 | 1.62 | 2.10E-06 | 1810 | 137 | 13588 | 2.19 | 7.14E-03 | 2.31E-04 | 3.84E-03 |
| GO:0034470~ncRNA processing | 43 | 1.74 | 5.99E-06 | 1810 | 158 | 13588 | 2.04 | 2.02E-02 | 6.18E-04 | 1.09E-02 |
| GO:0034660~ncRNA metabolic process | 50 | 2.03 | 1.64E-05 | 1810 | 202 | 13588 | 1.86 | 5.43E-02 | 1.55E-03 | 2.99E-02 |
| GO:0006364~rRNA processing | 25 | 1.01 | 1.79E-05 | 1810 | 74 | 13588 | 2.54 | 5.91E-02 | 1.60E-03 | 3.26E-02 |
| GO:0016072~rRNA metabolic process | 25 | 1.01 | 2.30E-05 | 1810 | 75 | 13588 | 2.50 | 7.53E-02 | 1.96E-03 | 4.20E-02 |
| GO:0042254~ribosome biogenesis | 30 | 1.22 | 2.58E-04 | 1810 | 112 | 13588 | 2.01 | 5.84E-01 | 1.45E-02 | 4.69E-01 |
| **Annotation Cluster 7**  **Enrichment Score: 3.6449508534078117** |  |  |  |  |  |  |  |  |  |  |
| GO:0009314~response to radiation | 39 | 1.58 | 1.65E-05 | 1810 | 143 | 13588 | 2.05 | 5.45E-02 | 1.51E-03 | 3.01E-02 |
| GO:0009416~response to light stimulus | 28 | 1.13 | 1.37E-04 | 1810 | 98 | 13588 | 2.14 | 3.74E-01 | 9.14E-03 | 2.51E-01 |
| GO:0009411~response to UV | 14 | 0.57 | 3.36E-04 | 1810 | 35 | 13588 | 3.00 | 6.82E-01 | 1.80E-02 | 6.12E-01 |
| GO:0009628~response to abiotic stimulus | 50 | 2.03 | 3.46E-03 | 1810 | 251 | 13588 | 1.50 | 1.00E+00 | 1.12E-01 | 6.13E+00 |
| **Annotation Cluster 8**  **Enrichment Score: 3.5311644757056033** |  |  |  |  |  |  |  |  |  |  |
| GO:0015986~ATP synthesis coupled proton transport | 19 | 0.77 | 2.26E-07 | 1810 | 37 | 13588 | 3.86 | 7.69E-04 | 3.08E-05 | 4.13E-04 |
| GO:0015985~energy coupled proton transport, down electrochemical gradient | 19 | 0.77 | 2.26E-07 | 1810 | 37 | 13588 | 3.86 | 7.69E-04 | 3.08E-05 | 4.13E-04 |
| GO:0006119~oxidative phosphorylation | 24 | 0.97 | 2.40E-07 | 1810 | 56 | 13588 | 3.22 | 8.17E-04 | 3.14E-05 | 4.38E-04 |
| GO:0034220~ion transmembrane transport | 19 | 0.77 | 2.33E-06 | 1810 | 42 | 13588 | 3.40 | 7.91E-03 | 2.48E-04 | 4.26E-03 |
| GO:0015992~proton transport | 20 | 0.81 | 2.00E-05 | 1810 | 52 | 13588 | 2.89 | 6.58E-02 | 1.74E-03 | 3.65E-02 |
| GO:0006818~hydrogen transport | 20 | 0.81 | 2.73E-05 | 1810 | 53 | 13588 | 2.83 | 8.88E-02 | 2.27E-03 | 4.98E-02 |
| GO:0009199~ribonucleoside triphosphate metabolic process | 29 | 1.18 | 1.12E-04 | 1810 | 102 | 13588 | 2.13 | 3.16E-01 | 8.06E-03 | 2.04E-01 |
| GO:0006754~ATP biosynthetic process | 25 | 1.01 | 1.15E-04 | 1810 | 82 | 13588 | 2.29 | 3.23E-01 | 8.09E-03 | 2.09E-01 |
| GO:0009201~ribonucleoside triphosphate biosynthetic process | 27 | 1.09 | 1.40E-04 | 1810 | 93 | 13588 | 2.18 | 3.79E-01 | 9.12E-03 | 2.55E-01 |
| GO:0009206~purine ribonucleoside triphosphate biosynthetic process | 27 | 1.09 | 1.40E-04 | 1810 | 93 | 13588 | 2.18 | 3.79E-01 | 9.12E-03 | 2.55E-01 |
| GO:0009145~purine nucleoside triphosphate biosynthetic process | 27 | 1.09 | 1.69E-04 | 1810 | 94 | 13588 | 2.16 | 4.38E-01 | 1.08E-02 | 3.09E-01 |
| GO:0009142~nucleoside triphosphate biosynthetic process | 27 | 1.09 | 2.04E-04 | 1810 | 95 | 13588 | 2.13 | 5.01E-01 | 1.26E-02 | 3.72E-01 |
| GO:0046034~ATP metabolic process | 26 | 1.05 | 2.08E-04 | 1810 | 90 | 13588 | 2.17 | 5.08E-01 | 1.26E-02 | 3.80E-01 |
| GO:0009205~purine ribonucleoside triphosphate metabolic process | 28 | 1.13 | 2.37E-04 | 1810 | 101 | 13588 | 2.08 | 5.54E-01 | 1.41E-02 | 4.32E-01 |
| GO:0009152~purine ribonucleotide biosynthetic process | 29 | 1.18 | 2.70E-04 | 1810 | 107 | 13588 | 2.03 | 6.02E-01 | 1.50E-02 | 4.92E-01 |
| GO:0009259~ribonucleotide metabolic process | 32 | 1.30 | 3.68E-04 | 1810 | 125 | 13588 | 1.92 | 7.14E-01 | 1.91E-02 | 6.69E-01 |
| GO:0009260~ribonucleotide biosynthetic process | 29 | 1.18 | 5.17E-04 | 1810 | 111 | 13588 | 1.96 | 8.28E-01 | 2.52E-02 | 9.39E-01 |
| GO:0009144~purine nucleoside triphosphate metabolic process | 28 | 1.13 | 5.47E-04 | 1810 | 106 | 13588 | 1.98 | 8.45E-01 | 2.63E-02 | 9.95E-01 |
| GO:0009150~purine ribonucleotide metabolic process | 30 | 1.22 | 7.59E-04 | 1810 | 119 | 13588 | 1.89 | 9.25E-01 | 3.48E-02 | 1.38E+00 |
| GO:0009141~nucleoside triphosphate metabolic process | 29 | 1.18 | 9.42E-04 | 1810 | 115 | 13588 | 1.89 | 9.60E-01 | 4.14E-02 | 1.71E+00 |
| GO:0006164~purine nucleotide biosynthetic process | 31 | 1.26 | 3.70E-03 | 1810 | 137 | 13588 | 1.70 | 1.00E+00 | 1.16E-01 | 6.54E+00 |
| GO:0006163~purine nucleotide metabolic process | 34 | 1.38 | 6.39E-03 | 1810 | 160 | 13588 | 1.60 | 1.00E+00 | 1.70E-01 | 1.11E+01 |
| GO:0044271~nitrogen compound biosynthetic process | 53 | 2.15 | 2.96E-02 | 1810 | 302 | 13588 | 1.32 | 1.00E+00 | 4.08E-01 | 4.22E+01 |
| GO:0034654~nucleobase, nucleoside, nucleotide and nucleic acid biosynthetic process | 32 | 1.30 | 7.34E-02 | 1810 | 179 | 13588 | 1.34 | 1.00E+00 | 6.21E-01 | 7.52E+01 |
| GO:0034404~nucleobase, nucleoside and nucleotide biosynthetic process | 32 | 1.30 | 7.34E-02 | 1810 | 179 | 13588 | 1.34 | 1.00E+00 | 6.21E-01 | 7.52E+01 |
| GO:0009165~nucleotide biosynthetic process | 31 | 1.26 | 8.08E-02 | 1810 | 174 | 13588 | 1.34 | 1.00E+00 | 6.39E-01 | 7.85E+01 |
| GO:0015672~monovalent inorganic cation transport | 49 | 1.99 | 1.12E-01 | 1810 | 303 | 13588 | 1.21 | 1.00E+00 | 7.31E-01 | 8.86E+01 |
| **Annotation Cluster 9**  **Enrichment Score: 3.5118625321004595** |  |  |  |  |  |  |  |  |  |  |
| GO:0006793~phosphorus metabolic process | 156 | 6.32 | 4.68E-05 | 1810 | 866 | 13588 | 1.35 | 1.47E-01 | 3.79E-03 | 8.54E-02 |
| GO:0006796~phosphate metabolic process | 156 | 6.32 | 4.68E-05 | 1810 | 866 | 13588 | 1.35 | 1.47E-01 | 3.79E-03 | 8.54E-02 |
| GO:0016310~phosphorylation | 129 | 5.23 | 2.51E-04 | 1810 | 718 | 13588 | 1.35 | 5.75E-01 | 1.44E-02 | 4.58E-01 |
| GO:0006468~protein amino acid phosphorylation | 105 | 4.26 | 1.63E-02 | 1810 | 640 | 13588 | 1.23 | 1.00E+00 | 3.00E-01 | 2.59E+01 |
| **Annotation Cluster 10**  **Enrichment Score: 2.8997235149375196** |  |  |  |  |  |  |  |  |  |  |
| GO:0006402~mRNA catabolic process | 16 | 0.65 | 1.98E-04 | 1810 | 42 | 13588 | 2.86 | 4.91E-01 | 1.24E-02 | 3.62E-01 |
| GO:0006401~RNA catabolic process | 16 | 0.65 | 1.01E-03 | 1810 | 48 | 13588 | 2.50 | 9.68E-01 | 4.26E-02 | 1.83E+00 |
| GO:0000956~nuclear-transcribed mRNA catabolic process | 12 | 0.49 | 2.63E-03 | 1810 | 33 | 13588 | 2.73 | 1.00E+00 | 9.29E-02 | 4.70E+00 |
| GO:0000184~nuclear-transcribed mRNA catabolic process, nonsense-mediated decay | 10 | 0.41 | 4.78E-03 | 1810 | 26 | 13588 | 2.89 | 1.00E+00 | 1.40E-01 | 8.38E+00 |
| **Annotation Cluster 11**  **Enrichment Score: 2.7123595217381244** |  |  |  |  |  |  |  |  |  |  |
| GO:0045333~cellular respiration | 19 | 0.77 | 4.56E-04 | 1810 | 59 | 13588 | 2.42 | 7.88E-01 | 2.29E-02 | 8.29E-01 |
| GO:0015980~energy derivation by oxidation of organic compounds | 25 | 1.01 | 1.94E-03 | 1810 | 98 | 13588 | 1.92 | 9.99E-01 | 7.56E-02 | 3.48E+00 |
| GO:0022904~respiratory electron transport chain | 10 | 0.41 | 8.26E-03 | 1810 | 28 | 13588 | 2.68 | 1.00E+00 | 2.07E-01 | 1.41E+01 |
| **Annotation Cluster 12**  **Enrichment Score: 2.669657409233663** |  |  |  |  |  |  |  |  |  |  |
| GO:0006302~double-strand break repair | 17 | 0.69 | 6.93E-05 | 1810 | 43 | 13588 | 2.97 | 2.10E-01 | 5.47E-03 | 1.26E-01 |
| GO:0000724~double-strand break repair via homologous recombination | 7 | 0.28 | 4.10E-03 | 1810 | 13 | 13588 | 4.04 | 1.00E+00 | 1.26E-01 | 7.23E+00 |
| GO:0000725~recombinational repair | 7 | 0.28 | 4.10E-03 | 1810 | 13 | 13588 | 4.04 | 1.00E+00 | 1.26E-01 | 7.23E+00 |
| GO:0006310~DNA recombination | 18 | 0.73 | 1.80E-02 | 1810 | 75 | 13588 | 1.80 | 1.00E+00 | 3.15E-01 | 2.82E+01 |
| **Annotation Cluster 13**  **Enrichment Score: 2.6228656192907023** |  |  |  |  |  |  |  |  |  |  |
| GO:0006350~transcription | 301 | 12.20 | 1.74E-06 | 1810 | 1772 | 13588 | 1.28 | 5.92E-03 | 2.05E-04 | 3.18E-03 |
| GO:0045449~regulation of transcription | 353 | 14.31 | 1.10E-04 | 1810 | 2227 | 13588 | 1.19 | 3.12E-01 | 8.10E-03 | 2.00E-01 |
| GO:0051252~regulation of RNA metabolic process | 203 | 8.23 | 3.90E-01 | 1810 | 1488 | 13588 | 1.02 | 1.00E+00 | 9.71E-01 | 1.00E+02 |
| GO:0006355~regulation of transcription, DNA-dependent | 198 | 8.03 | 4.32E-01 | 1810 | 1465 | 13588 | 1.01 | 1.00E+00 | 9.79E-01 | 1.00E+02 |
| **Annotation Cluster 14**  **Enrichment Score: 2.600308520499458** |  |  |  |  |  |  |  |  |  |  |
| GO:0022618~ribonucleoprotein complex assembly | 13 | 0.53 | 9.63E-04 | 1810 | 34 | 13588 | 2.87 | 9.62E-01 | 4.12E-02 | 1.74E+00 |
| GO:0000377~RNA splicing, via transesterification reactions with bulged adenosine as nucleophile | 13 | 0.53 | 2.22E-03 | 1810 | 37 | 13588 | 2.64 | 9.99E-01 | 8.24E-02 | 3.98E+00 |
| GO:0000375~RNA splicing, via transesterification reactions | 13 | 0.53 | 2.22E-03 | 1810 | 37 | 13588 | 2.64 | 9.99E-01 | 8.24E-02 | 3.98E+00 |
| GO:0000398~nuclear mRNA splicing, via spliceosome | 13 | 0.53 | 2.22E-03 | 1810 | 37 | 13588 | 2.64 | 9.99E-01 | 8.24E-02 | 3.98E+00 |
| GO:0000245~spliceosome assembly | 7 | 0.28 | 9.45E-03 | 1810 | 15 | 13588 | 3.50 | 1.00E+00 | 2.23E-01 | 1.59E+01 |
| **Annotation Cluster 15**  **Enrichment Score: 2.525805791160646** |  |  |  |  |  |  |  |  |  |  |
| GO:0034621~cellular macromolecular complex subunit organization | 55 | 2.23 | 1.05E-04 | 1810 | 245 | 13588 | 1.69 | 3.01E-01 | 7.93E-03 | 1.92E-01 |
| GO:0043933~macromolecular complex subunit organization | 72 | 2.92 | 6.51E-04 | 1810 | 367 | 13588 | 1.47 | 8.91E-01 | 3.03E-02 | 1.18E+00 |
| GO:0034622~cellular macromolecular complex assembly | 47 | 1.91 | 8.09E-04 | 1810 | 217 | 13588 | 1.63 | 9.37E-01 | 3.66E-02 | 1.47E+00 |
| GO:0065003~macromolecular complex assembly | 65 | 2.63 | 2.03E-03 | 1810 | 338 | 13588 | 1.44 | 9.99E-01 | 7.83E-02 | 3.65E+00 |
| GO:0070271~protein complex biogenesis | 39 | 1.58 | 7.88E-02 | 1810 | 227 | 13588 | 1.29 | 1.00E+00 | 6.32E-01 | 7.77E+01 |
| GO:0006461~protein complex assembly | 39 | 1.58 | 7.88E-02 | 1810 | 227 | 13588 | 1.29 | 1.00E+00 | 6.32E-01 | 7.77E+01 |
| **Annotation Cluster 16**  **Enrichment Score: 2.129207744958568** |  |  |  |  |  |  |  |  |  |  |
| GO:0006915~apoptosis | 84 | 3.40 | 2.85E-03 | 1810 | 465 | 13588 | 1.36 | 1.00E+00 | 9.83E-02 | 5.08E+00 |
| GO:0012501~programmed cell death | 84 | 3.40 | 4.59E-03 | 1810 | 473 | 13588 | 1.33 | 1.00E+00 | 1.37E-01 | 8.05E+00 |
| GO:0008219~cell death | 86 | 3.49 | 1.35E-02 | 1810 | 507 | 13588 | 1.27 | 1.00E+00 | 2.69E-01 | 2.20E+01 |
| GO:0016265~death | 87 | 3.53 | 1.72E-02 | 1810 | 519 | 13588 | 1.26 | 1.00E+00 | 3.08E-01 | 2.72E+01 |
| **Annotation Cluster 17**  **Enrichment Score: 2.0826515623325044** |  |  |  |  |  |  |  |  |  |  |
| GO:0042981~regulation of apoptosis | 103 | 4.18 | 3.12E-04 | 1810 | 553 | 13588 | 1.40 | 6.55E-01 | 1.70E-02 | 5.69E-01 |
| GO:0043067~regulation of programmed cell death | 103 | 4.18 | 4.96E-04 | 1810 | 560 | 13588 | 1.38 | 8.16E-01 | 2.46E-02 | 9.02E-01 |
| GO:0010941~regulation of cell death | 103 | 4.18 | 5.94E-04 | 1810 | 563 | 13588 | 1.37 | 8.68E-01 | 2.81E-02 | 1.08E+00 |
| GO:0043066~negative regulation of apoptosis | 49 | 1.99 | 2.10E-03 | 1810 | 239 | 13588 | 1.54 | 9.99E-01 | 7.89E-02 | 3.76E+00 |
| GO:0043069~negative regulation of programmed cell death | 49 | 1.99 | 3.23E-03 | 1810 | 244 | 13588 | 1.51 | 1.00E+00 | 1.07E-01 | 5.74E+00 |
| GO:0060548~negative regulation of cell death | 49 | 1.99 | 3.51E-03 | 1810 | 245 | 13588 | 1.50 | 1.00E+00 | 1.13E-01 | 6.23E+00 |
| GO:0043068~positive regulation of programmed cell death | 46 | 1.86 | 2.05E-02 | 1810 | 250 | 13588 | 1.38 | 1.00E+00 | 3.37E-01 | 3.16E+01 |
| GO:0010942~positive regulation of cell death | 46 | 1.86 | 2.33E-02 | 1810 | 252 | 13588 | 1.37 | 1.00E+00 | 3.60E-01 | 3.50E+01 |
| GO:0043065~positive regulation of apoptosis | 45 | 1.82 | 2.73E-02 | 1810 | 248 | 13588 | 1.36 | 1.00E+00 | 3.90E-01 | 3.97E+01 |
| GO:0006916~anti-apoptosis | 18 | 0.73 | 7.11E-02 | 1810 | 88 | 13588 | 1.54 | 1.00E+00 | 6.12E-01 | 7.40E+01 |
| GO:0012502~induction of programmed cell death | 27 | 1.09 | 2.23E-01 | 1810 | 167 | 13588 | 1.21 | 1.00E+00 | 8.90E-01 | 9.90E+01 |
| GO:0006917~induction of apoptosis | 27 | 1.09 | 2.23E-01 | 1810 | 167 | 13588 | 1.21 | 1.00E+00 | 8.90E-01 | 9.90E+01 |
| **Annotation Cluster 18**  **Enrichment Score: 1.973594044330888** |  |  |  |  |  |  |  |  |  |  |
| GO:0006631~fatty acid metabolic process | 38 | 1.54 | 6.24E-03 | 1810 | 184 | 13588 | 1.55 | 1.00E+00 | 1.69E-01 | 1.08E+01 |
| GO:0046394~carboxylic acid biosynthetic process | 30 | 1.22 | 1.05E-02 | 1810 | 141 | 13588 | 1.60 | 1.00E+00 | 2.34E-01 | 1.76E+01 |
| GO:0016053~organic acid biosynthetic process | 30 | 1.22 | 1.05E-02 | 1810 | 141 | 13588 | 1.60 | 1.00E+00 | 2.34E-01 | 1.76E+01 |
| GO:0006633~fatty acid biosynthetic process | 19 | 0.77 | 1.85E-02 | 1810 | 81 | 13588 | 1.76 | 1.00E+00 | 3.18E-01 | 2.89E+01 |
| **Annotation Cluster 19**  **Enrichment Score: 1.9140097717557996** |  |  |  |  |  |  |  |  |  |  |
| GO:0016126~sterol biosynthetic process | 12 | 0.49 | 1.09E-03 | 1810 | 30 | 13588 | 3.00 | 9.75E-01 | 4.52E-02 | 1.96E+00 |
| GO:0006695~cholesterol biosynthetic process | 10 | 0.41 | 1.81E-03 | 1810 | 23 | 13588 | 3.26 | 9.98E-01 | 7.18E-02 | 3.26E+00 |
| GO:0016125~sterol metabolic process | 19 | 0.77 | 1.11E-02 | 1810 | 77 | 13588 | 1.85 | 1.00E+00 | 2.40E-01 | 1.84E+01 |
| GO:0008203~cholesterol metabolic process | 17 | 0.69 | 1.97E-02 | 1810 | 70 | 13588 | 1.82 | 1.00E+00 | 3.31E-01 | 3.05E+01 |
| GO:0006694~steroid biosynthetic process | 16 | 0.65 | 4.47E-02 | 1810 | 71 | 13588 | 1.69 | 1.00E+00 | 5.09E-01 | 5.66E+01 |
| GO:0008202~steroid metabolic process | 27 | 1.09 | 1.70E-01 | 1810 | 161 | 13588 | 1.26 | 1.00E+00 | 8.39E-01 | 9.67E+01 |
| **Annotation Cluster 20**  **Enrichment Score: 1.899502718225913** |  |  |  |  |  |  |  |  |  |  |
| GO:0006913~nucleocytoplasmic transport | 27 | 1.09 | 2.45E-04 | 1810 | 96 | 13588 | 2.11 | 5.66E-01 | 1.43E-02 | 4.46E-01 |
| GO:0051169~nuclear transport | 27 | 1.09 | 3.49E-04 | 1810 | 98 | 13588 | 2.07 | 6.95E-01 | 1.84E-02 | 6.35E-01 |
| GO:0006605~protein targeting | 31 | 1.26 | 2.31E-03 | 1810 | 133 | 13588 | 1.75 | 1.00E+00 | 8.38E-02 | 4.14E+00 |
| GO:0006606~protein import into nucleus | 13 | 0.53 | 6.19E-02 | 1810 | 56 | 13588 | 1.74 | 1.00E+00 | 5.80E-01 | 6.89E+01 |
| GO:0017038~protein import | 17 | 0.69 | 7.27E-02 | 1810 | 82 | 13588 | 1.56 | 1.00E+00 | 6.19E-01 | 7.48E+01 |
| GO:0051170~nuclear import | 13 | 0.53 | 7.73E-02 | 1810 | 58 | 13588 | 1.68 | 1.00E+00 | 6.31E-01 | 7.70E+01 |
| GO:0033365~protein localization in organelle | 19 | 0.77 | 8.85E-02 | 1810 | 97 | 13588 | 1.47 | 1.00E+00 | 6.61E-01 | 8.16E+01 |
| GO:0034504~protein localization in nucleus | 13 | 0.53 | 1.05E-01 | 1810 | 61 | 13588 | 1.60 | 1.00E+00 | 7.15E-01 | 8.67E+01 |
| **Annotation Cluster 21**  **Enrichment Score: 1.8793913081085385** |  |  |  |  |  |  |  |  |  |  |
| GO:0006351~transcription, DNA-dependent | 28 | 1.13 | 4.90E-03 | 1810 | 122 | 13588 | 1.72 | 1.00E+00 | 1.42E-01 | 8.57E+00 |
| GO:0032774~RNA biosynthetic process | 28 | 1.13 | 7.68E-03 | 1810 | 126 | 13588 | 1.67 | 1.00E+00 | 1.98E-01 | 1.31E+01 |
| GO:0006366~transcription from RNA polymerase II promoter | 16 | 0.65 | 6.12E-02 | 1810 | 74 | 13588 | 1.62 | 1.00E+00 | 5.77E-01 | 6.84E+01 |
| **Annotation Cluster 22**  **Enrichment Score: 1.8412640315013413** |  |  |  |  |  |  |  |  |  |  |
| GO:0019320~hexose catabolic process | 16 | 0.65 | 2.47E-03 | 1810 | 52 | 13588 | 2.31 | 1.00E+00 | 8.84E-02 | 4.41E+00 |
| GO:0006007~glucose catabolic process | 16 | 0.65 | 2.47E-03 | 1810 | 52 | 13588 | 2.31 | 1.00E+00 | 8.84E-02 | 4.41E+00 |
| GO:0046365~monosaccharide catabolic process | 16 | 0.65 | 3.69E-03 | 1810 | 54 | 13588 | 2.22 | 1.00E+00 | 1.17E-01 | 6.52E+00 |
| GO:0006096~glycolysis | 14 | 0.57 | 3.73E-03 | 1810 | 44 | 13588 | 2.39 | 1.00E+00 | 1.16E-01 | 6.60E+00 |
| GO:0044275~cellular carbohydrate catabolic process | 16 | 0.65 | 1.05E-02 | 1810 | 60 | 13588 | 2.00 | 1.00E+00 | 2.35E-01 | 1.75E+01 |
| GO:0046164~alcohol catabolic process | 16 | 0.65 | 2.17E-02 | 1810 | 65 | 13588 | 1.85 | 1.00E+00 | 3.44E-01 | 3.30E+01 |
| GO:0019318~hexose metabolic process | 31 | 1.26 | 5.94E-02 | 1810 | 169 | 13588 | 1.38 | 1.00E+00 | 5.67E-01 | 6.73E+01 |
| GO:0016052~carbohydrate catabolic process | 17 | 0.69 | 6.63E-02 | 1810 | 81 | 13588 | 1.58 | 1.00E+00 | 5.96E-01 | 7.14E+01 |
| GO:0005996~monosaccharide metabolic process | 34 | 1.38 | 6.83E-02 | 1810 | 191 | 13588 | 1.34 | 1.00E+00 | 6.03E-01 | 7.25E+01 |
| GO:0006006~glucose metabolic process | 26 | 1.05 | 7.53E-02 | 1810 | 140 | 13588 | 1.39 | 1.00E+00 | 6.29E-01 | 7.61E+01 |
| **Annotation Cluster 23**  **Enrichment Score: 1.8002051245857542** |  |  |  |  |  |  |  |  |  |  |
| GO:0015936~coenzyme A metabolic process | 6 | 0.24 | 5.86E-03 | 1810 | 10 | 13588 | 4.50 | 1.00E+00 | 1.64E-01 | 1.02E+01 |
| GO:0015937~coenzyme A biosynthetic process | 5 | 0.20 | 7.84E-03 | 1810 | 7 | 13588 | 5.36 | 1.00E+00 | 2.00E-01 | 1.34E+01 |
| GO:0033865~nucleoside bisphosphate metabolic process | 6 | 0.24 | 9.58E-03 | 1810 | 11 | 13588 | 4.09 | 1.00E+00 | 2.23E-01 | 1.61E+01 |
| GO:0042278~purine nucleoside metabolic process | 9 | 0.36 | 9.90E-03 | 1810 | 24 | 13588 | 2.82 | 1.00E+00 | 2.26E-01 | 1.66E+01 |
| GO:0046128~purine ribonucleoside metabolic process | 9 | 0.36 | 9.90E-03 | 1810 | 24 | 13588 | 2.82 | 1.00E+00 | 2.26E-01 | 1.66E+01 |
| GO:0009119~ribonucleoside metabolic process | 11 | 0.45 | 2.83E-02 | 1810 | 39 | 13588 | 2.12 | 1.00E+00 | 3.96E-01 | 4.08E+01 |
| GO:0009116~nucleoside metabolic process | 11 | 0.45 | 2.05E-01 | 1810 | 56 | 13588 | 1.47 | 1.00E+00 | 8.75E-01 | 9.85E+01 |
| **Annotation Cluster 24**  **Enrichment Score: 1.7940261723517494** |  |  |  |  |  |  |  |  |  |  |
| GO:0031575~G1/S transition checkpoint | 6 | 0.24 | 5.86E-03 | 1810 | 10 | 13588 | 4.50 | 1.00E+00 | 1.64E-01 | 1.02E+01 |
| GO:0042770~DNA damage response, signal transduction | 16 | 0.65 | 6.39E-03 | 1810 | 57 | 13588 | 2.11 | 1.00E+00 | 1.72E-01 | 1.11E+01 |
| GO:0007093~mitotic cell cycle checkpoint | 9 | 0.36 | 9.90E-03 | 1810 | 24 | 13588 | 2.82 | 1.00E+00 | 2.26E-01 | 1.66E+01 |
| GO:0000077~DNA damage checkpoint | 10 | 0.41 | 1.34E-02 | 1810 | 30 | 13588 | 2.50 | 1.00E+00 | 2.70E-01 | 2.18E+01 |
| GO:0000075~cell cycle checkpoint | 14 | 0.57 | 1.66E-02 | 1810 | 52 | 13588 | 2.02 | 1.00E+00 | 2.99E-01 | 2.63E+01 |
| GO:0031570~DNA integrity checkpoint | 10 | 0.41 | 2.50E-02 | 1810 | 33 | 13588 | 2.27 | 1.00E+00 | 3.71E-01 | 3.70E+01 |
| GO:0031571~G1 DNA damage checkpoint | 3 | 0.12 | 1.35E-01 | 1810 | 5 | 13588 | 4.50 | 1.00E+00 | 7.81E-01 | 9.29E+01 |
| **Annotation Cluster 25**  **Enrichment Score: 1.7578569172701928** |  |  |  |  |  |  |  |  |  |  |
| GO:0006487~protein amino acid N-linked glycosylation | 9 | 0.36 | 1.29E-02 | 1810 | 25 | 13588 | 2.70 | 1.00E+00 | 2.63E-01 | 2.11E+01 |
| GO:0043413~biopolymer glycosylation | 21 | 0.85 | 1.52E-02 | 1810 | 91 | 13588 | 1.73 | 1.00E+00 | 2.91E-01 | 2.44E+01 |
| GO:0006486~protein amino acid glycosylation | 21 | 0.85 | 1.52E-02 | 1810 | 91 | 13588 | 1.73 | 1.00E+00 | 2.91E-01 | 2.44E+01 |
| GO:0070085~glycosylation | 21 | 0.85 | 1.52E-02 | 1810 | 91 | 13588 | 1.73 | 1.00E+00 | 2.91E-01 | 2.44E+01 |
| GO:0009100~glycoprotein metabolic process | 30 | 1.22 | 1.83E-02 | 1810 | 147 | 13588 | 1.53 | 1.00E+00 | 3.19E-01 | 2.86E+01 |
| GO:0009101~glycoprotein biosynthetic process | 24 | 0.97 | 3.41E-02 | 1810 | 117 | 13588 | 1.54 | 1.00E+00 | 4.41E-01 | 4.69E+01 |
| **Annotation Cluster 26**  **Enrichment Score: 1.7551528019397613** |  |  |  |  |  |  |  |  |  |  |
| GO:0032268~regulation of cellular protein metabolic process | 54 | 2.19 | 4.73E-03 | 1810 | 280 | 13588 | 1.45 | 1.00E+00 | 1.40E-01 | 8.29E+00 |
| GO:0006417~regulation of translation | 22 | 0.89 | 2.14E-02 | 1810 | 100 | 13588 | 1.65 | 1.00E+00 | 3.44E-01 | 3.27E+01 |
| GO:0010608~posttranscriptional regulation of gene expression | 28 | 1.13 | 5.35E-02 | 1810 | 148 | 13588 | 1.42 | 1.00E+00 | 5.44E-01 | 6.34E+01 |
| **Annotation Cluster 27**  **Enrichment Score: 1.739628962711035** |  |  |  |  |  |  |  |  |  |  |
| GO:0051053~negative regulation of DNA metabolic process | 9 | 0.36 | 2.76E-03 | 1810 | 20 | 13588 | 3.38 | 1.00E+00 | 9.64E-02 | 4.93E+00 |
| GO:0051052~regulation of DNA metabolic process | 17 | 0.69 | 2.97E-03 | 1810 | 58 | 13588 | 2.20 | 1.00E+00 | 1.00E-01 | 5.29E+00 |
| GO:0006275~regulation of DNA replication | 7 | 0.28 | 2.47E-02 | 1810 | 18 | 13588 | 2.92 | 1.00E+00 | 3.69E-01 | 3.66E+01 |
| GO:0008156~negative regulation of DNA replication | 5 | 0.20 | 4.77E-02 | 1810 | 11 | 13588 | 3.41 | 1.00E+00 | 5.24E-01 | 5.90E+01 |
| GO:0000018~regulation of DNA recombination | 6 | 0.24 | 2.07E-01 | 1810 | 24 | 13588 | 1.88 | 1.00E+00 | 8.77E-01 | 9.86E+01 |
| **Annotation Cluster 28**  **Enrichment Score: 1.6643456678556197** |  |  |  |  |  |  |  |  |  |  |
| GO:0030705~cytoskeleton-dependent intracellular transport | 11 | 0.45 | 8.64E-03 | 1810 | 33 | 13588 | 2.50 | 1.00E+00 | 2.11E-01 | 1.47E+01 |
| GO:0010970~microtubule-based transport | 7 | 0.28 | 2.47E-02 | 1810 | 18 | 13588 | 2.92 | 1.00E+00 | 3.69E-01 | 3.66E+01 |
| GO:0008088~axon cargo transport | 5 | 0.20 | 4.77E-02 | 1810 | 11 | 13588 | 3.41 | 1.00E+00 | 5.24E-01 | 5.90E+01 |
| **Annotation Cluster 29**  **Enrichment Score: 1.652234512218465** |  |  |  |  |  |  |  |  |  |  |
| GO:0010469~regulation of receptor activity | 6 | 0.24 | 5.86E-03 | 1810 | 10 | 13588 | 4.50 | 1.00E+00 | 1.64E-01 | 1.02E+01 |
| GO:0007176~regulation of epidermal growth factor receptor activity | 5 | 0.20 | 2.26E-02 | 1810 | 9 | 13588 | 4.17 | 1.00E+00 | 3.53E-01 | 3.42E+01 |
| GO:0042058~regulation of epidermal growth factor receptor signaling pathway | 5 | 0.20 | 8.33E-02 | 1810 | 13 | 13588 | 2.89 | 1.00E+00 | 6.45E-01 | 7.96E+01 |
| **Annotation Cluster 30**  **Enrichment Score: 1.6467885085599705** |  |  |  |  |  |  |  |  |  |  |
| GO:0006732~coenzyme metabolic process | 31 | 1.26 | 7.05E-03 | 1810 | 143 | 13588 | 1.63 | 1.00E+00 | 1.85E-01 | 1.21E+01 |
| GO:0051186~cofactor metabolic process | 37 | 1.50 | 9.00E-03 | 1810 | 182 | 13588 | 1.53 | 1.00E+00 | 2.17E-01 | 1.52E+01 |
| GO:0051188~cofactor biosynthetic process | 19 | 0.77 | 5.32E-02 | 1810 | 91 | 13588 | 1.57 | 1.00E+00 | 5.43E-01 | 6.32E+01 |
| GO:0009108~coenzyme biosynthetic process | 14 | 0.57 | 7.66E-02 | 1810 | 64 | 13588 | 1.64 | 1.00E+00 | 6.31E-01 | 7.67E+01 |
| **Annotation Cluster 31**  **Enrichment Score: 1.5728394520149203** |  |  |  |  |  |  |  |  |  |  |
| GO:0007254~JNK cascade | 10 | 0.41 | 2.05E-02 | 1810 | 32 | 13588 | 2.35 | 1.00E+00 | 3.39E-01 | 3.15E+01 |
| GO:0000165~MAPKKK cascade | 24 | 0.97 | 2.59E-02 | 1810 | 114 | 13588 | 1.58 | 1.00E+00 | 3.77E-01 | 3.81E+01 |
| GO:0031098~stress-activated protein kinase signaling pathway | 10 | 0.41 | 3.59E-02 | 1810 | 35 | 13588 | 2.14 | 1.00E+00 | 4.55E-01 | 4.87E+01 |
| **Annotation Cluster 32**  **Enrichment Score: 1.567182810872196** |  |  |  |  |  |  |  |  |  |  |
| GO:0048167~regulation of synaptic plasticity | 14 | 0.57 | 8.34E-03 | 1810 | 48 | 13588 | 2.19 | 1.00E+00 | 2.06E-01 | 1.42E+01 |
| GO:0048168~regulation of neuronal synaptic plasticity | 9 | 0.36 | 2.08E-02 | 1810 | 27 | 13588 | 2.50 | 1.00E+00 | 3.39E-01 | 3.19E+01 |
| GO:0050804~regulation of synaptic transmission | 22 | 0.89 | 2.14E-02 | 1810 | 100 | 13588 | 1.65 | 1.00E+00 | 3.44E-01 | 3.27E+01 |
| GO:0031644~regulation of neurological system process | 24 | 0.97 | 2.36E-02 | 1810 | 113 | 13588 | 1.59 | 1.00E+00 | 3.62E-01 | 3.53E+01 |
| GO:0051969~regulation of transmission of nerve impulse | 23 | 0.93 | 2.37E-02 | 1810 | 107 | 13588 | 1.61 | 1.00E+00 | 3.62E-01 | 3.55E+01 |
| GO:0048169~regulation of long-term neuronal synaptic plasticity | 7 | 0.28 | 3.21E-02 | 1810 | 19 | 13588 | 2.77 | 1.00E+00 | 4.28E-01 | 4.49E+01 |
| GO:0044057~regulation of system process | 33 | 1.34 | 1.60E-01 | 1810 | 201 | 13588 | 1.23 | 1.00E+00 | 8.22E-01 | 9.59E+01 |
| **Annotation Cluster 33**  **Enrichment Score: 1.5433366226208214** |  |  |  |  |  |  |  |  |  |  |
| GO:0007049~cell cycle | 120 | 4.86 | 8.21E-06 | 1810 | 611 | 13588 | 1.47 | 2.76E-02 | 8.22E-04 | 1.50E-02 |
| GO:0022402~cell cycle process | 74 | 3.00 | 1.75E-03 | 1810 | 393 | 13588 | 1.41 | 9.97E-01 | 7.03E-02 | 3.15E+00 |
| GO:0022403~cell cycle phase | 58 | 2.35 | 2.00E-02 | 1810 | 328 | 13588 | 1.33 | 1.00E+00 | 3.33E-01 | 3.09E+01 |
| GO:0000279~M phase | 48 | 1.95 | 6.25E-02 | 1810 | 283 | 13588 | 1.27 | 1.00E+00 | 5.80E-01 | 6.92E+01 |
| GO:0000278~mitotic cell cycle | 42 | 1.70 | 6.70E-02 | 1810 | 244 | 13588 | 1.29 | 1.00E+00 | 5.98E-01 | 7.18E+01 |
| GO:0051301~cell division | 47 | 1.91 | 7.84E-02 | 1810 | 281 | 13588 | 1.26 | 1.00E+00 | 6.32E-01 | 7.75E+01 |
| GO:0048285~organelle fission | 31 | 1.26 | 2.41E-01 | 1810 | 197 | 13588 | 1.18 | 1.00E+00 | 9.03E-01 | 9.93E+01 |
| GO:0000280~nuclear division | 30 | 1.22 | 2.41E-01 | 1810 | 190 | 13588 | 1.19 | 1.00E+00 | 9.03E-01 | 9.94E+01 |
| GO:0007067~mitosis | 30 | 1.22 | 2.41E-01 | 1810 | 190 | 13588 | 1.19 | 1.00E+00 | 9.03E-01 | 9.94E+01 |
| GO:0000087~M phase of mitotic cell cycle | 30 | 1.22 | 2.79E-01 | 1810 | 194 | 13588 | 1.16 | 1.00E+00 | 9.28E-01 | 9.97E+01 |
| **Annotation Cluster 34**  **Enrichment Score: 1.4669305463019922** |  |  |  |  |  |  |  |  |  |  |
| GO:0009890~negative regulation of biosynthetic process | 76 | 3.08 | 9.85E-03 | 1810 | 434 | 13588 | 1.31 | 1.00E+00 | 2.27E-01 | 1.65E+01 |
| GO:0010558~negative regulation of macromolecule biosynthetic process | 73 | 2.96 | 1.21E-02 | 1810 | 418 | 13588 | 1.31 | 1.00E+00 | 2.54E-01 | 1.99E+01 |
| GO:0010605~negative regulation of macromolecule metabolic process | 86 | 3.49 | 1.29E-02 | 1810 | 506 | 13588 | 1.28 | 1.00E+00 | 2.65E-01 | 2.10E+01 |
| GO:0051172~negative regulation of nitrogen compound metabolic process | 70 | 2.84 | 1.40E-02 | 1810 | 401 | 13588 | 1.31 | 1.00E+00 | 2.75E-01 | 2.28E+01 |
| GO:0031327~negative regulation of cellular biosynthetic process | 74 | 3.00 | 1.59E-02 | 1810 | 430 | 13588 | 1.29 | 1.00E+00 | 2.97E-01 | 2.54E+01 |
| GO:0045934~negative regulation of nucleobase, nucleoside, nucleotide and nucleic acid metabolic process | 69 | 2.80 | 1.62E-02 | 1810 | 397 | 13588 | 1.30 | 1.00E+00 | 3.00E-01 | 2.58E+01 |
| GO:0010629~negative regulation of gene expression | 69 | 2.80 | 3.07E-02 | 1810 | 410 | 13588 | 1.26 | 1.00E+00 | 4.17E-01 | 4.34E+01 |
| GO:0016481~negative regulation of transcription | 62 | 2.51 | 4.74E-02 | 1810 | 372 | 13588 | 1.25 | 1.00E+00 | 5.24E-01 | 5.88E+01 |
| GO:0000122~negative regulation of transcription from RNA polymerase II promoter | 37 | 1.50 | 1.76E-01 | 1810 | 231 | 13588 | 1.20 | 1.00E+00 | 8.45E-01 | 9.71E+01 |
| GO:0045892~negative regulation of transcription, DNA-dependent | 47 | 1.91 | 2.20E-01 | 1810 | 308 | 13588 | 1.15 | 1.00E+00 | 8.88E-01 | 9.89E+01 |
| GO:0051253~negative regulation of RNA metabolic process | 47 | 1.91 | 2.34E-01 | 1810 | 310 | 13588 | 1.14 | 1.00E+00 | 8.99E-01 | 9.92E+01 |
| **Annotation Cluster 35**  **Enrichment Score: 1.4187884322049782** |  |  |  |  |  |  |  |  |  |  |
| GO:0031175~neuron projection development | 43 | 1.74 | 8.13E-03 | 1810 | 218 | 13588 | 1.48 | 1.00E+00 | 2.05E-01 | 1.38E+01 |
| GO:0032990~cell part morphogenesis | 42 | 1.70 | 8.28E-03 | 1810 | 212 | 13588 | 1.49 | 1.00E+00 | 2.06E-01 | 1.41E+01 |
| GO:0048667~cell morphogenesis involved in neuron differentiation | 36 | 1.46 | 1.51E-02 | 1810 | 182 | 13588 | 1.48 | 1.00E+00 | 2.91E-01 | 2.43E+01 |
| GO:0048858~cell projection morphogenesis | 39 | 1.58 | 1.65E-02 | 1810 | 202 | 13588 | 1.45 | 1.00E+00 | 3.01E-01 | 2.62E+01 |
| GO:0000904~cell morphogenesis involved in differentiation | 40 | 1.62 | 2.14E-02 | 1810 | 212 | 13588 | 1.42 | 1.00E+00 | 3.43E-01 | 3.27E+01 |
| GO:0048666~neuron development | 52 | 2.11 | 2.44E-02 | 1810 | 292 | 13588 | 1.34 | 1.00E+00 | 3.69E-01 | 3.64E+01 |
| GO:0007409~axonogenesis | 32 | 1.30 | 2.45E-02 | 1810 | 163 | 13588 | 1.47 | 1.00E+00 | 3.68E-01 | 3.64E+01 |
| GO:0030030~cell projection organization | 56 | 2.27 | 2.53E-02 | 1810 | 319 | 13588 | 1.32 | 1.00E+00 | 3.73E-01 | 3.74E+01 |
| GO:0048812~neuron projection morphogenesis | 33 | 1.34 | 3.99E-02 | 1810 | 176 | 13588 | 1.41 | 1.00E+00 | 4.84E-01 | 5.25E+01 |
| GO:0030182~neuron differentiation | 65 | 2.63 | 6.27E-02 | 1810 | 399 | 13588 | 1.22 | 1.00E+00 | 5.80E-01 | 6.93E+01 |
| GO:0000902~cell morphogenesis | 51 | 2.07 | 8.09E-02 | 1810 | 309 | 13588 | 1.24 | 1.00E+00 | 6.38E-01 | 7.86E+01 |
| GO:0032989~cellular component morphogenesis | 56 | 2.27 | 1.10E-01 | 1810 | 351 | 13588 | 1.20 | 1.00E+00 | 7.27E-01 | 8.80E+01 |
| GO:0007411~axon guidance | 18 | 0.73 | 1.51E-01 | 1810 | 98 | 13588 | 1.38 | 1.00E+00 | 8.12E-01 | 9.50E+01 |
| GO:0006928~cell motion | 46 | 1.86 | 7.48E-01 | 1810 | 367 | 13588 | 0.94 | 1.00E+00 | 9.99E-01 | 1.00E+02 |
| **Annotation Cluster 36**  **Enrichment Score: 1.310490592853516** |  |  |  |  |  |  |  |  |  |  |
| GO:0006744~ubiquinone biosynthetic process | 5 | 0.20 | 2.26E-02 | 1810 | 9 | 13588 | 4.17 | 1.00E+00 | 3.53E-01 | 3.42E+01 |
| GO:0006743~ubiquinone metabolic process | 5 | 0.20 | 2.26E-02 | 1810 | 9 | 13588 | 4.17 | 1.00E+00 | 3.53E-01 | 3.42E+01 |
| GO:0045426~quinone cofactor biosynthetic process | 5 | 0.20 | 3.38E-02 | 1810 | 10 | 13588 | 3.75 | 1.00E+00 | 4.40E-01 | 4.66E+01 |
| GO:0009108~coenzyme biosynthetic process | 14 | 0.57 | 7.66E-02 | 1810 | 64 | 13588 | 1.64 | 1.00E+00 | 6.31E-01 | 7.67E+01 |
| GO:0042375~quinone cofactor metabolic process | 5 | 0.20 | 8.33E-02 | 1810 | 13 | 13588 | 2.89 | 1.00E+00 | 6.45E-01 | 7.96E+01 |
| GO:0006733~oxidoreduction coenzyme metabolic process | 9 | 0.36 | 1.24E-01 | 1810 | 38 | 13588 | 1.78 | 1.00E+00 | 7.60E-01 | 9.11E+01 |
