# Supplementary material for: Genome-Wide Histone Acetylation Is Altered in a Transgenic Mouse Model of Huntington's Disease
Source: PLoS One. 2012 Jul 27;7(7):e41423. doi: 10.1371/journal.pone.0041423 (PMC3407195; doi:10.1371/journal.pone.0041423)
Supplement: Table S4 — Gene Ontology (GO)-Biological Process (GOTERM_BP_FAT) Functional Annotation Clustering of “Not acetylated in TG” genes. (DOCX) [file pone.0041423.s004.docx]

Supplemental Table 4: Gene Ontology (GO)-Biological Process (GOTERM_BP_FAT) Functional Annotation Clustering of “Not acetylated in TG” genes

| **Term** | **Count** | **%** | **PValue** | **List Total** | **Pop Hits** | **Pop Total** | **Fold Enrichment** | **Bonferroni** | **Benjamini** | **FDR** |
| --- | --- | --- | --- | --- | --- | --- | --- | --- | --- | --- |
| **Annotation Cluster 1**  **Enrichment Score: 5.94546419206833** | | | | | | | | | | |
| GO:0008104~protein localization | 116 | 6.66 | 1.85E-08 | 1245 | 753 | 13588 | 1.68 | 5.54E-05 | 5.54E-05 | 3.33E-05 |
| GO:0015031~protein transport | 102 | 5.86 | 6.54E-08 | 1245 | 651 | 13588 | 1.71 | 1.96E-04 | 9.79E-05 | 1.18E-04 |
| GO:0045184~establishment of protein localization | 102 | 5.86 | 9.74E-08 | 1245 | 656 | 13588 | 1.70 | 2.92E-04 | 9.73E-05 | 1.75E-04 |
| GO:0006886~intracellular protein transport | 52 | 2.99 | 8.52E-07 | 1245 | 276 | 13588 | 2.06 | 2.55E-03 | 5.11E-04 | 1.53E-03 |
| GO:0034613~cellular protein localization | 54 | 3.10 | 1.95E-06 | 1245 | 299 | 13588 | 1.97 | 5.83E-03 | 9.74E-04 | 3.51E-03 |
| GO:0070727~cellular macromolecule localization | 54 | 3.10 | 2.39E-06 | 1245 | 301 | 13588 | 1.96 | 7.14E-03 | 8.96E-04 | 4.31E-03 |
| GO:0046907~intracellular transport | 70 | 4.02 | 2.77E-06 | 1245 | 431 | 13588 | 1.77 | 8.25E-03 | 9.20E-04 | 4.98E-03 |
| GO:0006605~protein targeting | 24 | 1.38 | 2.11E-03 | 1245 | 133 | 13588 | 1.97 | 9.98E-01 | 1.70E-01 | 3.73E+00 |
| **Annotation Cluster 2**  **Enrichment Score: 3.8814492658466566** | | | | | | | | | | |
| GO:0006793~phosphorus metabolic process | 113 | 6.49 | 9.17E-05 | 1245 | 866 | 13588 | 1.42 | 2.40E-01 | 1.70E-02 | 1.65E-01 |
| GO:0006796~phosphate metabolic process | 113 | 6.49 | 9.17E-05 | 1245 | 866 | 13588 | 1.42 | 2.40E-01 | 1.70E-02 | 1.65E-01 |
| GO:0016310~phosphorylation | 96 | 5.51 | 1.33E-04 | 1245 | 718 | 13588 | 1.46 | 3.29E-01 | 2.32E-02 | 2.40E-01 |
| GO:0006468~protein amino acid phosphorylation | 86 | 4.94 | 2.66E-04 | 1245 | 640 | 13588 | 1.47 | 5.49E-01 | 3.56E-02 | 4.78E-01 |
| **Annotation Cluster 3**  **Enrichment Score: 3.6712186091427497** | | | | | | | | | | |
| GO:0006396~RNA processing | 67 | 3.85 | 3.12E-05 | 1245 | 437 | 13588 | 1.67 | 8.94E-02 | 7.77E-03 | 5.62E-02 |
| GO:0016071~mRNA metabolic process | 50 | 2.87 | 5.33E-05 | 1245 | 302 | 13588 | 1.81 | 1.48E-01 | 1.22E-02 | 9.60E-02 |
| GO:0006397~mRNA processing | 42 | 2.41 | 4.59E-04 | 1245 | 262 | 13588 | 1.75 | 7.47E-01 | 5.35E-02 | 8.22E-01 |
| GO:0008380~RNA splicing | 32 | 1.84 | 2.70E-03 | 1245 | 201 | 13588 | 1.74 | 1.00E+00 | 1.97E-01 | 4.76E+00 |
| **Annotation Cluster 4**  **Enrichment Score: 3.3196647948005618** | | | | | | | | | | |
| GO:0009057~macromolecule catabolic process | 90 | 5.17 | 8.16E-05 | 1245 | 654 | 13588 | 1.50 | 2.17E-01 | 1.73E-02 | 1.47E-01 |
| GO:0030163~protein catabolic process | 79 | 4.54 | 8.33E-05 | 1245 | 556 | 13588 | 1.55 | 2.21E-01 | 1.65E-02 | 1.50E-01 |
| GO:0044265~cellular macromolecule catabolic process | 84 | 4.82 | 1.33E-04 | 1245 | 609 | 13588 | 1.51 | 3.30E-01 | 2.20E-02 | 2.40E-01 |
| GO:0044257~cellular protein catabolic process | 75 | 4.31 | 2.22E-04 | 1245 | 537 | 13588 | 1.52 | 4.86E-01 | 3.12E-02 | 3.99E-01 |
| GO:0051603~proteolysis involved in cellular protein catabolic process | 74 | 4.25 | 3.05E-04 | 1245 | 534 | 13588 | 1.51 | 6.00E-01 | 3.90E-02 | 5.49E-01 |
| GO:0019941~modification-dependent protein catabolic process | 70 | 4.02 | 5.36E-04 | 1245 | 508 | 13588 | 1.50 | 7.99E-01 | 5.78E-02 | 9.61E-01 |
| GO:0043632~modification-dependent macromolecule catabolic process | 70 | 4.02 | 5.36E-04 | 1245 | 508 | 13588 | 1.50 | 7.99E-01 | 5.78E-02 | 9.61E-01 |
| GO:0006508~proteolysis | 105 | 6.03 | 1.57E-01 | 1245 | 1034 | 13588 | 1.11 | 1.00E+00 | 9.18E-01 | 9.54E+01 |
| **Annotation Cluster 5**  **Enrichment Score: 2.8326996076299817** | | | | | | | | | | |
| GO:0007010~cytoskeleton organization | 54 | 3.10 | 2.51E-05 | 1245 | 326 | 13588 | 1.81 | 7.26E-02 | 6.82E-03 | 4.52E-02 |
| GO:0030029~actin filament-based process | 30 | 1.72 | 1.32E-03 | 1245 | 176 | 13588 | 1.86 | 9.81E-01 | 1.20E-01 | 2.35E+00 |
| GO:0030036~actin cytoskeleton organization | 28 | 1.61 | 2.11E-03 | 1245 | 165 | 13588 | 1.85 | 9.98E-01 | 1.74E-01 | 3.73E+00 |
| GO:0007015~actin filament organization | 10 | 0.57 | 6.66E-02 | 1245 | 56 | 13588 | 1.95 | 1.00E+00 | 7.94E-01 | 7.11E+01 |
| **Annotation Cluster 6**  **Enrichment Score: 2.656704339624215** | | | | | | | | | | |
| GO:0006399~tRNA metabolic process | 24 | 1.38 | 1.99E-04 | 1245 | 113 | 13588 | 2.32 | 4.49E-01 | 3.09E-02 | 3.57E-01 |
| GO:0043039~tRNA aminoacylation | 13 | 0.75 | 6.75E-04 | 1245 | 46 | 13588 | 3.08 | 8.68E-01 | 6.98E-02 | 1.21E+00 |
| GO:0043038~amino acid activation | 13 | 0.75 | 6.75E-04 | 1245 | 46 | 13588 | 3.08 | 8.68E-01 | 6.98E-02 | 1.21E+00 |
| GO:0006418~tRNA aminoacylation for protein translation | 13 | 0.75 | 6.75E-04 | 1245 | 46 | 13588 | 3.08 | 8.68E-01 | 6.98E-02 | 1.21E+00 |
| GO:0034660~ncRNA metabolic process | 33 | 1.89 | 1.50E-03 | 1245 | 202 | 13588 | 1.78 | 9.89E-01 | 1.31E-01 | 2.67E+00 |
| GO:0008033~tRNA processing | 12 | 0.69 | 5.24E-02 | 1245 | 70 | 13588 | 1.87 | 1.00E+00 | 7.63E-01 | 6.21E+01 |
| GO:0034470~ncRNA processing | 22 | 1.26 | 5.25E-02 | 1245 | 158 | 13588 | 1.52 | 1.00E+00 | 7.61E-01 | 6.22E+01 |
| **Annotation Cluster 7**  **Enrichment Score: 1.999079955907064** |  |  |  |  |  |  |  |  |  |  |
| GO:0006333~chromatin assembly or disassembly | 20 | 1.15 | 4.51E-03 | 1245 | 109 | 13588 | 2.00 | 1.00E+00 | 2.93E-01 | 7.81E+00 |
| GO:0006325~chromatin organization | 44 | 2.53 | 5.11E-03 | 1245 | 315 | 13588 | 1.52 | 1.00E+00 | 3.19E-01 | 8.80E+00 |
| GO:0051276~chromosome organization | 51 | 2.93 | 1.70E-02 | 1245 | 404 | 13588 | 1.38 | 1.00E+00 | 5.70E-01 | 2.66E+01 |
| GO:0016568~chromatin modification | 32 | 1.84 | 2.57E-02 | 1245 | 236 | 13588 | 1.48 | 1.00E+00 | 6.47E-01 | 3.74E+01 |
| **Annotation Cluster 8**  **Enrichment Score: 1.8203784291946394** | | | | | | | | | | |
| GO:0044092~negative regulation of molecular function | 25 | 1.44 | 8.34E-04 | 1245 | 132 | 13588 | 2.07 | 9.18E-01 | 7.99E-02 | 1.49E+00 |
| GO:0043086~negative regulation of catalytic activity | 19 | 1.09 | 4.42E-03 | 1245 | 101 | 13588 | 2.05 | 1.00E+00 | 2.95E-01 | 7.66E+00 |
| GO:0051348~negative regulation of transferase activity | 11 | 0.63 | 2.05E-02 | 1245 | 53 | 13588 | 2.27 | 1.00E+00 | 6.04E-01 | 3.11E+01 |
| GO:0051338~regulation of transferase activity | 28 | 1.61 | 2.47E-02 | 1245 | 199 | 13588 | 1.54 | 1.00E+00 | 6.47E-01 | 3.63E+01 |
| GO:0043549~regulation of kinase activity | 27 | 1.55 | 2.77E-02 | 1245 | 192 | 13588 | 1.53 | 1.00E+00 | 6.65E-01 | 3.97E+01 |
| GO:0045859~regulation of protein kinase activity | 26 | 1.49 | 3.29E-02 | 1245 | 186 | 13588 | 1.53 | 1.00E+00 | 6.85E-01 | 4.53E+01 |
| GO:0033673~negative regulation of kinase activity | 10 | 0.57 | 4.01E-02 | 1245 | 51 | 13588 | 2.14 | 1.00E+00 | 7.18E-01 | 5.21E+01 |
| GO:0006469~negative regulation of protein kinase activity | 10 | 0.57 | 4.01E-02 | 1245 | 51 | 13588 | 2.14 | 1.00E+00 | 7.18E-01 | 5.21E+01 |
| **Annotation Cluster 9**  **Enrichment Score: 1.6862616680484583** | | | | | | | | | | |
| GO:0006626~protein targeting to mitochondrion | 7 | 0.40 | 1.20E-02 | 1245 | 22 | 13588 | 3.47 | 1.00E+00 | 4.83E-01 | 1.96E+01 |
| GO:0070585~protein localization in mitochondrion | 7 | 0.40 | 1.20E-02 | 1245 | 22 | 13588 | 3.47 | 1.00E+00 | 4.83E-01 | 1.96E+01 |
| GO:0007005~mitochondrion organization | 17 | 0.98 | 1.46E-02 | 1245 | 97 | 13588 | 1.91 | 1.00E+00 | 5.32E-01 | 2.32E+01 |
| GO:0033365~protein localization in organelle | 16 | 0.92 | 3.01E-02 | 1245 | 97 | 13588 | 1.80 | 1.00E+00 | 6.73E-01 | 4.23E+01 |
| GO:0006839~mitochondrial transport | 9 | 0.52 | 3.44E-02 | 1245 | 42 | 13588 | 2.34 | 1.00E+00 | 6.93E-01 | 4.68E+01 |
| GO:0017038~protein import | 14 | 0.80 | 3.48E-02 | 1245 | 82 | 13588 | 1.86 | 1.00E+00 | 6.93E-01 | 4.72E+01 |
| **Annotation Cluster 10**  **Enrichment Score: 1.582525346609846** | | | | | | | | | | |
| GO:0006351~transcription, DNA-dependent | 21 | 1.21 | 7.33E-03 | 1245 | 122 | 13588 | 1.88 | 1.00E+00 | 4.01E-01 | 1.24E+01 |
| GO:0032774~RNA biosynthetic process | 21 | 1.21 | 1.04E-02 | 1245 | 126 | 13588 | 1.82 | 1.00E+00 | 4.47E-01 | 1.72E+01 |
| GO:0006366~transcription from RNA polymerase II promoter | 10 | 0.57 | 2.34E-01 | 1245 | 74 | 13588 | 1.47 | 1.00E+00 | 9.56E-01 | 9.92E+01 |
| **Annotation Cluster 11**  **Enrichment Score: 1.5073166400495202** | | | | | | | | | | |
| GO:0006333~chromatin assembly or disassembly | 20 | 1.15 | 4.51E-03 | 1245 | 109 | 13588 | 2.00 | 1.00E+00 | 2.93E-01 | 7.81E+00 |
| GO:0065004~protein-DNA complex assembly | 15 | 0.86 | 8.44E-03 | 1245 | 76 | 13588 | 2.15 | 1.00E+00 | 4.24E-01 | 1.41E+01 |
| GO:0006334~nucleosome assembly | 14 | 0.80 | 1.44E-02 | 1245 | 73 | 13588 | 2.09 | 1.00E+00 | 5.33E-01 | 2.30E+01 |
| GO:0031497~chromatin assembly | 14 | 0.80 | 1.78E-02 | 1245 | 75 | 13588 | 2.04 | 1.00E+00 | 5.75E-01 | 2.76E+01 |
| GO:0034728~nucleosome organization | 14 | 0.80 | 1.97E-02 | 1245 | 76 | 13588 | 2.01 | 1.00E+00 | 6.01E-01 | 3.02E+01 |
| GO:0006323~DNA packaging | 14 | 0.80 | 1.32E-01 | 1245 | 101 | 13588 | 1.51 | 1.00E+00 | 9.02E-01 | 9.21E+01 |
| GO:0034621~cellular macromolecular complex subunit organization | 28 | 1.61 | 1.80E-01 | 1245 | 245 | 13588 | 1.25 | 1.00E+00 | 9.34E-01 | 9.72E+01 |
| GO:0034622~cellular macromolecular complex assembly | 25 | 1.44 | 1.92E-01 | 1245 | 217 | 13588 | 1.26 | 1.00E+00 | 9.42E-01 | 9.78E+01 |
| **Annotation Cluster 12**  **Enrichment Score: 1.4027525821775555** | | | | | | | | | | |
| GO:0070646~protein modification by small protein removal | 7 | 0.40 | 2.26E-02 | 1245 | 25 | 13588 | 3.06 | 1.00E+00 | 6.29E-01 | 3.37E+01 |
| GO:0070647~protein modification by small protein conjugation or removal | 16 | 0.92 | 5.13E-02 | 1245 | 104 | 13588 | 1.68 | 1.00E+00 | 7.62E-01 | 6.12E+01 |
| GO:0016579~protein deubiquitination | 6 | 0.34 | 5.34E-02 | 1245 | 23 | 13588 | 2.85 | 1.00E+00 | 7.64E-01 | 6.28E+01 |
| **Annotation Cluster 13**  **Enrichment Score: 1.3549422226397012** | | | | | | | | | | |
| GO:0051236~establishment of RNA localization | 12 | 0.69 | 3.62E-02 | 1245 | 66 | 13588 | 1.98 | 1.00E+00 | 6.99E-01 | 4.85E+01 |
| GO:0050657~nucleic acid transport | 12 | 0.69 | 3.62E-02 | 1245 | 66 | 13588 | 1.98 | 1.00E+00 | 6.99E-01 | 4.85E+01 |
| GO:0050658~RNA transport | 12 | 0.69 | 3.62E-02 | 1245 | 66 | 13588 | 1.98 | 1.00E+00 | 6.99E-01 | 4.85E+01 |
| GO:0006403~RNA localization | 12 | 0.69 | 3.99E-02 | 1245 | 67 | 13588 | 1.95 | 1.00E+00 | 7.19E-01 | 5.19E+01 |
| GO:0051028~mRNA transport | 11 | 0.63 | 5.39E-02 | 1245 | 62 | 13588 | 1.94 | 1.00E+00 | 7.61E-01 | 6.31E+01 |
| GO:0015931~nucleobase, nucleoside, nucleotide and nucleic acid transport | 12 | 0.69 | 7.29E-02 | 1245 | 74 | 13588 | 1.77 | 1.00E+00 | 8.11E-01 | 7.44E+01 |
