# Supplementary material for: Genome-Wide Histone Acetylation Is Altered in a Transgenic Mouse Model of Huntington's Disease
Source: PLoS One. 2012 Jul 27;7(7):e41423. doi: 10.1371/journal.pone.0041423 (PMC3407195; doi:10.1371/journal.pone.0041423)
Supplement: Table S5 — Gene Ontology (GO)-Biological Process(GOTERM_BP_FAT) Functional Annotation Clustering of “Hyperacetylated in TG” genes. (DOCX) [file pone.0041423.s005.docx]

Supplemental Table 5: Gene Ontology (GO)-Biological Process(GOTERM_BP_FAT) Functional Annotation Clustering of “Hyperacetylated in TG” genes

| **Term** | **Count** | **%** | **PValue** | **List Total** | **Pop Hits** | **Pop Total** | **Fold Enrichment** | **Bonferroni** | **Benjamini** | **FDR** |
| --- | --- | --- | --- | --- | --- | --- | --- | --- | --- | --- |
| **Annotation Cluster 1**  **Enrichment Score: 1.6127838520704079** | | | | | | | | | | |
| GO:0006350~transcription | 25 | 17.48 | 1.96E-03 | 101 | 1772 | 13588 | 1.90 | 7.68E-01 | 7.68E-01 | 2.94E+00 |
| GO:0045449~regulation of transcription | 28 | 19.58 | 4.79E-03 | 101 | 2227 | 13588 | 1.69 | 9.72E-01 | 8.33E-01 | 7.04E+00 |
| GO:0006355~regulation of transcription, DNA-dependent | 15 | 10.49 | 1.87E-01 | 101 | 1465 | 13588 | 1.38 | 1.00E+00 | 9.57E-01 | 9.57E+01 |
| GO:0051252~regulation of RNA metabolic process | 15 | 10.49 | 2.02E-01 | 101 | 1488 | 13588 | 1.36 | 1.00E+00 | 9.53E-01 | 9.68E+01 |
| **Annotation Cluster 2**  **Enrichment Score: 1.3652728546551314** | | | | | | | | | | |
| GO:0015031~protein transport | 12 | 8.39 | 8.27E-03 | 101 | 651 | 13588 | 2.48 | 9.98E-01 | 7.87E-01 | 1.19E+01 |
| GO:0045184~establishment of protein localization | 12 | 8.39 | 8.73E-03 | 101 | 656 | 13588 | 2.46 | 9.99E-01 | 7.29E-01 | 1.25E+01 |
| GO:0008104~protein localization | 13 | 9.09 | 9.15E-03 | 101 | 753 | 13588 | 2.32 | 9.99E-01 | 6.81E-01 | 1.30E+01 |
| GO:0017038~protein import | 4 | 2.80 | 2.25E-02 | 101 | 82 | 13588 | 6.56 | 1.00E+00 | 9.12E-01 | 2.93E+01 |
| GO:0033365~protein localization in organelle | 4 | 2.80 | 3.47E-02 | 101 | 97 | 13588 | 5.55 | 1.00E+00 | 9.46E-01 | 4.15E+01 |
| GO:0046907~intracellular transport | 8 | 5.59 | 3.96E-02 | 101 | 431 | 13588 | 2.50 | 1.00E+00 | 9.51E-01 | 4.59E+01 |
| GO:0006886~intracellular protein transport | 6 | 4.20 | 5.30E-02 | 101 | 276 | 13588 | 2.92 | 1.00E+00 | 9.33E-01 | 5.63E+01 |
| GO:0006606~protein import into nucleus | 3 | 2.10 | 6.39E-02 | 101 | 56 | 13588 | 7.21 | 1.00E+00 | 9.35E-01 | 6.34E+01 |
| GO:0051170~nuclear import | 3 | 2.10 | 6.80E-02 | 101 | 58 | 13588 | 6.96 | 1.00E+00 | 9.37E-01 | 6.57E+01 |
| GO:0034613~cellular protein localization | 6 | 4.20 | 6.97E-02 | 101 | 299 | 13588 | 2.70 | 1.00E+00 | 9.32E-01 | 6.66E+01 |
| GO:0070727~cellular macromolecule localization | 6 | 4.20 | 7.12E-02 | 101 | 301 | 13588 | 2.68 | 1.00E+00 | 9.18E-01 | 6.75E+01 |
| GO:0034504~protein localization in nucleus | 3 | 2.10 | 7.42E-02 | 101 | 61 | 13588 | 6.62 | 1.00E+00 | 9.18E-01 | 6.90E+01 |
| GO:0006605~protein targeting | 4 | 2.80 | 7.49E-02 | 101 | 133 | 13588 | 4.05 | 1.00E+00 | 9.11E-01 | 6.94E+01 |
| GO:0006913~nucleocytoplasmic transport | 3 | 2.10 | 1.57E-01 | 101 | 96 | 13588 | 4.20 | 1.00E+00 | 9.55E-01 | 9.26E+01 |
| GO:0051169~nuclear transport | 3 | 2.10 | 1.62E-01 | 101 | 98 | 13588 | 4.12 | 1.00E+00 | 9.57E-01 | 9.33E+01 |
