# Supplementary material for: Genome-Wide Histone Acetylation Is Altered in a Transgenic Mouse Model of Huntington's Disease
Source: PLoS One. 2012 Jul 27;7(7):e41423. doi: 10.1371/journal.pone.0041423 (PMC3407195; doi:10.1371/journal.pone.0041423)
Supplement: Table S6 — Gene Ontology (GO)-Biological Process (GOTERM_BP_FAT) Functional Annotation Clustering of “Ectopically acetylated in TG” genes. (DOCX) [file pone.0041423.s006.docx]

Supplemental Table 6: Gene Ontology (GO)-Biological Process (GOTERM_BP_FAT) Functional Annotation Clustering of “Ectopically acetylated in TG” genes

| **Term** | **Count** | **%** | **PValue** | **List Total** | **Pop Hits** | **Pop Total** | **Fold Enrichment** | **Bonferroni** | **Benjamini** | **FDR** |
| --- | --- | --- | --- | --- | --- | --- | --- | --- | --- | --- |
| **Annotation Cluster**  **Enrichment Score: 1.3637692603679046** | | | | | | | | | | |
| GO:0008406~gonad development | 9 | 1.84 | 2.84E-03 | 374 | 88 | 13588 | 3.72 | 9.95E-01 | 9.28E-01 | 4.73E+00 |
| GO:0045137~development of primary sexual characteristics | 9 | 1.84 | 7.82E-03 | 374 | 104 | 13588 | 3.14 | 1.00E+00 | 8.36E-01 | 1.25E+01 |
| GO:0022602~ovulation cycle process | 6 | 1.22 | 9.04E-03 | 374 | 47 | 13588 | 4.64 | 1.00E+00 | 8.13E-01 | 1.43E+01 |
| GO:0042698~ovulation cycle | 6 | 1.22 | 9.87E-03 | 374 | 48 | 13588 | 4.54 | 1.00E+00 | 8.10E-01 | 1.55E+01 |
| GO:0008585~female gonad development | 6 | 1.22 | 1.48E-02 | 374 | 53 | 13588 | 4.11 | 1.00E+00 | 8.80E-01 | 2.24E+01 |
| GO:0046545~development of primary female sexual characteristics | 6 | 1.22 | 1.98E-02 | 374 | 57 | 13588 | 3.82 | 1.00E+00 | 9.00E-01 | 2.88E+01 |
| GO:0007548~sex differentiation | 9 | 1.84 | 2.69E-02 | 374 | 130 | 13588 | 2.52 | 1.00E+00 | 9.09E-01 | 3.71E+01 |
| GO:0048608~reproductive structure development | 9 | 1.84 | 2.69E-02 | 374 | 130 | 13588 | 2.52 | 1.00E+00 | 9.09E-01 | 3.71E+01 |
| GO:0046660~female sex differentiation | 6 | 1.22 | 3.09E-02 | 374 | 64 | 13588 | 3.41 | 1.00E+00 | 9.28E-01 | 4.14E+01 |
| GO:0001541~ovarian follicle development | 4 | 0.82 | 6.11E-02 | 374 | 33 | 13588 | 4.40 | 1.00E+00 | 9.33E-01 | 6.58E+01 |
| GO:0008584~male gonad development | 4 | 0.82 | 9.09E-02 | 374 | 39 | 13588 | 3.73 | 1.00E+00 | 9.52E-01 | 8.03E+01 |
| GO:0048609~reproductive process in a multicellular organism | 17 | 3.47 | 9.62E-02 | 374 | 409 | 13588 | 1.51 | 1.00E+00 | 9.55E-01 | 8.21E+01 |
| GO:0032504~multicellular organism reproduction | 17 | 3.47 | 9.62E-02 | 374 | 409 | 13588 | 1.51 | 1.00E+00 | 9.55E-01 | 8.21E+01 |
| GO:0048511~rhythmic process | 6 | 1.22 | 1.32E-01 | 374 | 98 | 13588 | 2.22 | 1.00E+00 | 9.59E-01 | 9.11E+01 |
| GO:0046546~development of primary male sexual characteristics | 4 | 0.82 | 1.78E-01 | 374 | 53 | 13588 | 2.74 | 1.00E+00 | 9.72E-01 | 9.64E+01 |
| GO:0003006~reproductive developmental process | 11 | 2.24 | 1.92E-01 | 374 | 264 | 13588 | 1.51 | 1.00E+00 | 9.70E-01 | 9.74E+01 |
| GO:0046661~male sex differentiation | 4 | 0.82 | 2.06E-01 | 374 | 57 | 13588 | 2.55 | 1.00E+00 | 9.70E-01 | 9.80E+01 |
| GO:0007292~female gamete generation | 3 | 0.61 | 4.57E-01 | 374 | 56 | 13588 | 1.95 | 1.00E+00 | 9.94E-01 | 1.00E+02 |
| **Annotation Cluster 2**  **Enrichment Score: 1.357867376943978** | | | | | | | | | | |
| GO:0048870~cell motility | 15 | 3.06 | 2.57E-02 | 374 | 284 | 13588 | 1.92 | 1.00E+00 | 9.09E-01 | 3.58E+01 |
| GO:0051674~localization of cell | 15 | 3.06 | 2.57E-02 | 374 | 284 | 13588 | 1.92 | 1.00E+00 | 9.09E-01 | 3.58E+01 |
| GO:0016477~cell migration | 12 | 2.45 | 6.82E-02 | 374 | 240 | 13588 | 1.82 | 1.00E+00 | 9.48E-01 | 7.00E+01 |
| GO:0006928~cell motion | 16 | 3.27 | 8.24E-02 | 374 | 367 | 13588 | 1.58 | 1.00E+00 | 9.53E-01 | 7.69E+01 |
